# Reconstructing the maize leaf regulatory network using ChIP-seq data of 104 transcription factors

**DOI:** 10.1038/s41467-020-18832-8

**Published:** 2020-10-09

**Authors:** Xiaoyu Tu, María Katherine Mejía-Guerra, Jose A. Valdes Franco, David Tzeng, Po-Yu Chu, Wei Shen, Yingying Wei, Xiuru Dai, Pinghua Li, Edward S. Buckler, Silin Zhong

**Affiliations:** 1 The State Key Laboratory of Crop Biology, College of Agronomy, Shandong Agricultural University, Shandong, China; 2 State Key Laboratory of Agrobiotechnology, School of Life Sciences, The Chinese University of Hong Kong, Hong Kong, China; 3 Institute for Genomic Diversity, Cornell University, Ithaca, NY USA; 4 Department of Statistics, The Chinese University of Hong Kong, Hong Kong, China; 5 School of Integrative Plant Sciences, Section of Plant Breeding and Genetics, Cornell University, Ithaca, NY USA; 6 Agricultural Research Service, United States Department of Agriculture, Ithaca, NY USA

**Keywords:** Transcriptomics, Gene regulatory networks, Gene expression, Plant molecular biology

## Abstract

The transcription regulatory network inside a eukaryotic cell is defined by the combinatorial actions of transcription factors (TFs). However, TF binding studies in plants are too few in number to produce a general picture of this complex network. In this study, we use large-scale ChIP-seq to reconstruct it in the maize leaf, and train machine-learning models to predict TF binding and co-localization. The resulting network covers 77% of the expressed genes, and shows a scale-free topology and functional modularity like a real-world network. TF binding sequence preferences are conserved within family, while co-binding could be key for their binding specificity. Cross-species comparison shows that core network nodes at the top of the transmission of information being more conserved than those at the bottom. This study reveals the complex and redundant nature of the plant transcription regulatory network, and sheds light on its architecture, organizing principle and evolutionary trajectory.

## Abstract (web summary)

Transcriptional factors (TFs) bind in a combinatorial fashion to specify the on-and-off states of genes in a complex and redundant regulatory network. Here, the authors construct the transcription regulatory network in maize leaf using 104 TFs ChIP-seq data and train machine learning models to predict TF binding and colocalization.

## Introduction

For all living cells, a basic control task is to determine gene expression throughout time and space during developmental processes and in responses to stimuli^1^. The code that governs gene expression is stored in the noncoding region of the genome. Sequence-specific DNA binding proteins known as transcription factors (TFs) can read the code by binding to *cis*-regulatory elements to activate or repress gene expression. Each gene receives instructions from multiple TFs, and each TF targets thousands of genes, forming a regulatory network, which controls almost all biological processes inside the cell^2^. Hence, it is critical to understand the mechanism, architecture and behavior, as well as conservation and diversification of the regulatory network.

The budding yeast has probably the best mapped transcription regulatory network and is a unicellular model for eukaryotes, with ~200 TF coding genes^3,4^. For multicellular organisms with large genomes and thousands of TFs, complete network reconstruction has proven to be a formidable task. For example, the ENCODE consortium has mapped the binding of 88 TFs in 5 cell lines to study its architecture and dynamics^5^, while a more complete regulatory network was constructed in human colorectal cancer cells with data from 112 TFs^6^. Similar efforts are seldom feasible for individual laboratories, and have yet to be attempted in plants.

Maize is one of the best-studied and most tractable genetic system among the cereal crops, making it an ideal model for studying this group of important plants^7^. It has been shown that the maize noncoding region is a major contributor to the observable phenotypic differences and adaptation, between and within species. For example, 70% of maize genome-wide association study (GWAS) hits are located in the noncoding regions without functional annotation^8^. Sequence variations in the open chromatin regions, which often harbor TF-binding sites, could explain up to 40% of the phenotypic variations of key maize agronomic traits^9^. However, it is difficult to assess their true functions without knowing the precise locations of the *cis*-regulatory elements and the TFs that recognize them.

Despite TF-binding information being crucial for understanding how genes are regulated, ChIP-seq experiments in plants are often limited by antibody availability and difficulties in transforming crops to express the epitope fusion proteins. As a result, ChIP-seq data is only available for a handful of maize TFs, and even in the model species like Arabidopsis and rice, such studies are too few in number to produce a general picture of the plant transcription regulatory network.

In this study, we carry out ChIP-seq for 104 TFs that are expressed in the maize leaf, reconstruct its transcription regulatory network, and train machine-learning models to predict TF binding and co-localization. Our findings reveal the architecture, organizing principle and evolution of the plant transcription regulatory network, and provide a valuable resource for understanding how biological processes are regulated in leaf.

## Results

### Large-scale TF ChIP-seq using maize leaf protoplast

**Fig. 1:**
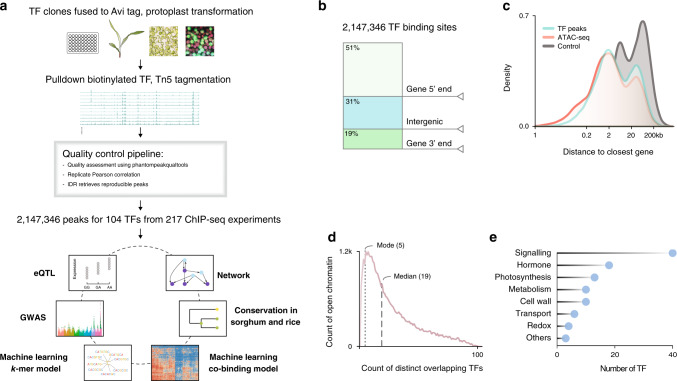
The transcription regulatory landscape of the maize leaf. **a** Overview of the experimental and analytical approaches. **b** Genome-wide distribution of TF-binding loci. **c** Density plots corresponding to distances of TF-binding sites (green) and open chromatin regions (red) to closest annotated gene. Gene-to-gene distant is used as a control (gray). **d** Distribution of the number of TFs-binding sites in open chromatin region. **e** Functional classification of the 104 TF based on their target genes. The height of the bar indicates the number of TF associated with each MapMan category.

We have developed an efficient protoplast isolation and transformation system to express epitope tagged TFs for large-scale ChIP-seq (Fig. 1 and Supplementary Method 1). We found that the high mortality rate and low transformation efficiency associated with the conventional methods are due to excessive wounding during cell wall digestion. Instead of cutting the leaf, we peeled the lower epidermis to allow the cell wall digestion enzymes to gently penetrate the leaf and release the protoplasts. Using this method, we could obtain ~10^7^ intact mesophyll protoplasts from the leaves of two 9-day-old seedlings, and achieve over 90% transformation efficiency. In addition, we fused the TF coding sequence to the Avi epitope tag, which can be biotinylated by the co-expressed biotin ligase. The strong and specific interaction between biotin and streptavidin magnetic beads enabled us to obtain high signal-to-noise ratio in difficult ChIP-seq experiment with limited starting materials. To reduce library construction cost, we generated a hyper-stable Tn5 transposase fused to the C-terminal of the *E. coli* elongation factor Ts, which could be easily purified for tagmentation ChIP-seq library preparation. To obtain large quantities of DNA for transformation, we developed a plasmid preparation protocol using low cost sand powder as binding matrix and detergent to remove bacterial endotoxin without expensive cation exchange resin.

Using the maize protoplast system, we have successfully performed over 200 ChIP-seq experiments for 104 TFs that are expressed in the developing section of the maize leaf, based on previous RNA-seq data^10^ (Supplementary Data 1). We then applied the ENCODE2 uniform pipeline to process the ChIP-seq data^11^. In total, 217 ChIP-seq experiments have passed the quality control with normalized strand cross-correlation and relative strand cross-correlation no less than 1.05 and 0.8, respectively (Supplementary Datas 2 and 3). After checking the reproducibility between biological replicates (Pearson correlation ≥ 0.8, Supplementary Fig. 1; Supplementary Data 4), peaks were then called with the ENCODE2 statistical framework (irreproducible discovery rate ≤ 0.01). We identified a total of 2,147,346 reproducible TF-binding peaks, and the number of peaks varies between TFs, with a median value around 16,000 binding sites per TF (IQR_25–75_: 7664–32,566).

We anticipated that plant TF binding might form dense clusters and frequently locate within open chromatin regions based on previous mammalian studies^6^. To test this, we merged all TF-binding peaks in the genome and found that they indeed clustered into 144,890 non-overlapping loci, covering ~2% of the genome (Supplementary Data 5). Similar saturation of TF-binding clusters was observed when sufficiently large number of TFs were examined in colorectal cancer cells^6^. Next, we measured chromatin accessibility in the same tissue using ATAC-seq, and found that the TF-binding loci and open chromatin regions showed similar genome-wide distributions, frequently proximal to gene bodies (±2.5 kb), with preferences for the 5′ end (Fig. 1b, c). The distance between TF-binding site and gene, after excluding regions that overlap with the gene body, is bimodal. Despite the larger space available for distal regulation, we observed that regions between 10 and 100 kb constitute ~15% of the TF-binding loci, and ~17% of the open chromatin regions (Fig. 1c). Layering ATAC-seq and ChIP-seq data showed that TF-binding loci and open chromatin overlap (Supplementary Fig. 1d; *P* value < 10^−5^). On average, ~74% of the peaks for a given TF (IQR_25–75_: 64–87%) intersect with open chromatin regions confirming the relevance of the identified TF-binding sites within the chromatin context (Supplementary Fig. 1e). Collectively, 98% of the open chromatin regions overlap with TF peaks (Fig. 1d), with a mode of 5, and a median of 19 distinct TFs for each region, suggesting there are a large number of possible TF combinations that coregulate transcription, in contrast to the classic view of a singular or few regulators that control the expression of a gene.

As none of the 104 TFs have been previously examined by ChIP-seq, and most of them have not been functionally characterized, we used GO-term and MAPMAN functional category enrichment analysis to classify them based on their target genes (Fig. 1e and Supplementary Data 6). The majority of the TFs are grouped into signaling, hormone, photosynthesis, and metabolism categories, which are the core biological functions of the leaf. For example, target genes of the maize ZIM TFs show enrichment in the GO-terms response to wounding and jasmonic acid metabolism, consistent with the role of their homologs in other plant species^12^. ZmMYB38 is a known regulator of the flavonoid pathway, and can directly bind to the ZmCOMT1 gene^13^. As expected, the targets of ZmMYB38 were associated with terms such as regulation of flavonoid biosynthetic process and phenylpropanoid biosynthetic process, and were assigned to the metabolic group.

**Fig. 2:**
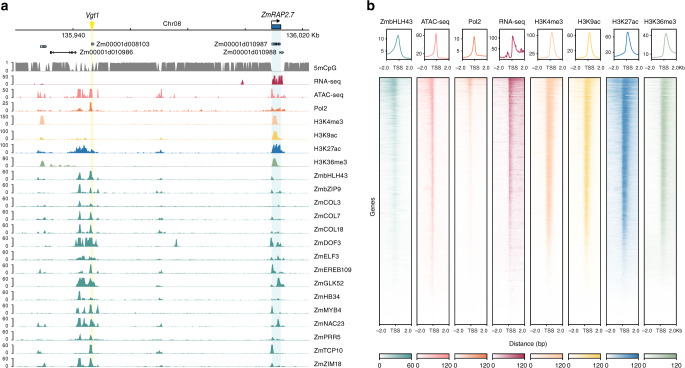
TF binding in the *Vgt1* locus. **a** Genome browser tracks showing the *ZmRAP2.7* gene and its distal regulatory region *Vgt1*. The DNA methylation in CG context, RNA-seq, ATAC-seq, chromatin marks, as well as binding of TFs from different families are shown. **b** Heatmaps and signal density plots centered on transcriptional start sites of genes targeted by ZmbHLH43, one of the TFs that bind to *Vgt1*.

Previous studies showed that the high TF occupancy regions in the genome are often associated with important functions^14,15^. We identified 2037 open chromatin regions in the top 5% of the TF occupancy distribution, and their surrounding genes are indeed enriched for regulatory GO-terms (Supplementary Data 7). Notably, we observed that a distal regulatory region *Vgt1*, an important QTL for flowering time^16^, is a high TF occupancy region bound by 76 TFs. This region is located at a distance of ~72 kb from the *ZmRAP2.7*, whose expression is regulated by *Vgt1* (Fig. 2a). Six of the TFs bound to this distal region (i.e., PRR5, ELF3, COL3, COL7, COL18, and DOF3/PBF1) have been previously linked to flowering time variations through genetic studies^17,18^. Although over half of the TF binding sites are located at the gene 5′ proximal region (Fig. 1b), distal ones such as *Vgt1* also show similar chromatin signatures and could play an important role in regulating transcription (Fig. 2; Supplementary Fig. 2).

### Sequence conservation and functional enrichment in TF-binding loci

**Fig. 3:**
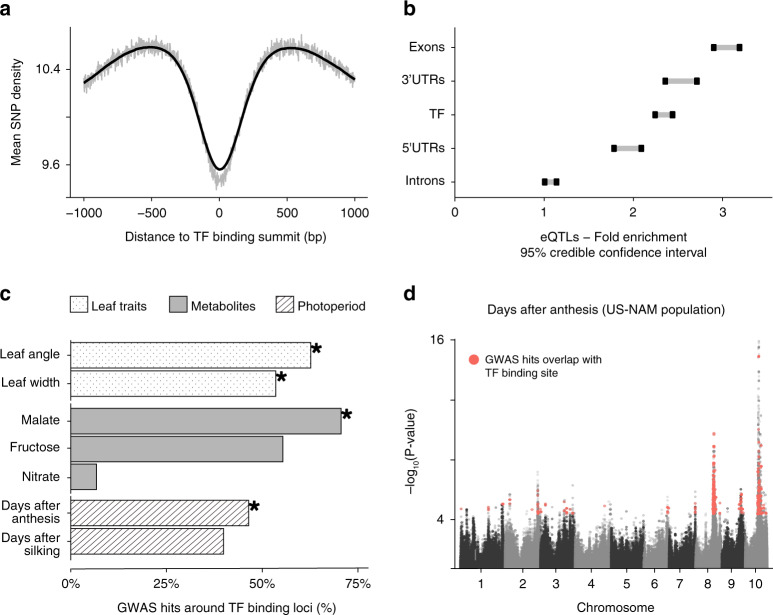
TF-binding sites show low sequence diversity and enrichment in functional variation. **a** Distribution of the average SNP count in maize inbred lines around TF peaks (bin size: 100 bp). The smooth line corresponds to a sliding window of 100 positions to average the mean SNP counts. **b** 95% confidence interval for the enrichment of eQTLs from a previous study^21^ vs. non-eQTLs SNPs, and relative to control regions for different sets of genomic regions (TF *n* = 144,890, exons *n* = 403,199, 3′ UTR *n* = 138,793, 5′ UTR *n* = 146,120, introns *n* = 151,414). **c** Proportion of phenotype-associated GWAS hits for an assortment of traits overlapping to TF-binding loci. Traits in which the enrichment was statistically significant (adj-*P* < 0.05) are labeled with an asterisk. **d** Manhattan plot of GWAS for days after anthesis. GWAS hits overlap with binding regions for a group of TFs previously associated with photoperiod variation are highlighted in red. Source data underlying Fig. 3c are provided in a Source Data file.

If the identified TF-binding loci are key to transcriptional regulation, and purifying selection is effective in these regions, they should exhibit low sequence diversity. We examined the conservation of the TF-binding sites by assessing the overall nucleotide diversity represented in the maize HapMap^19^ while controlling for overall single-nucleotide polymorphism (SNP) density in function to the distance of TF’s peak summit (Fig. 3a). The result confirmed that sequence variation in these regions is, in fact, reduced, suggesting that they could have important regulatory functions.

While binding of most TFs is constrained, TFs and their binding sites are also key to local adaptation or domestication. For example, ZmTB1 has been shown to play an important role in maize domestication^20^. Hence, we predict that TF-binding loci could be enriched for common SNP variations controlling gene expression and downstream traits. This was firstly tested in a panel of 282 inbred breeding lines for their effect on mRNA expression using common and likely adaptive variants^21^. We found twofold enrichment of TF-binding loci (95% credible interval: 2.26–2.46), similar to the enrichment around 5′ and 3′ UTRs (Fig. 3b). Distal and proximal TF binding loci were also enriched when examined separately (Supplementary Fig. 3a). The enrichment pattern is ubiquitous across the 104 TFs that we studied (Supplementary Fig. 3b and Supplementary Data 8). Overall, these results support our hypothesis that common variation in mRNA expression is controlled by both proximal and distal TF-binding site variations.

We also tested the overlap of TF-binding sites with functional variations associated with agricultural traits other than gene expression. To this end, we calculated the enrichment in GWAS hits for seven traits related to metabolites^22^, leaf architecture^23^, and photoperiodicity^24^ measured in the US NAM population (Supplementary Data 9). Overall, TF-binding loci are enriched for four of the traits (Fig. 3c). We noticed that simple traits, such as metabolites, showed few TFs enriched for GWAS hits (e.g., malate and nitrate). In contrast, many TF-binding sites overlap with GWAS hits for complex traits, which are known to be polygenic and influenced by a large number of genetic variants (e.g., days after silking and days after anthesis; Fig. 3d and Supplementary Fig. 3c). A further look at TFs enriched in GWAS hits for photoperiodicity revealed that 51% of the TFs enriched in days after anthesis, and 35% in days after silking, also bound to the *Vgt1*/*ZmRAP2.7* region, suggesting that they could be potential regulators of maize flowering time.

Overall, we found that TF-binding sites are conserved and frequently overlap with sequence variations associated with phenotypic changes. The general trend for GWAS enrichment in these regions also supports our hypothesis that noncoding region variations related to complex traits are mediated by TFs. Furthermore, our finding highlighted the potential of using TF ChIP-seq data to connect sequence variation in *cis* to *trans* regulators to highlight the molecular mechanism implicated in complex phenotypes.

### A scale-free transcription regulatory network with TF genes as hubs

Next, we constructed a gene regulatory network using the ENCODE TIP probabilistic framework, which identifies TF-target genes based on high-confidence proximal interactions^5,11,25^. To assess the feasibility of using our data to pinpoint true regulatory relationships, we overlaid our result with those inferred by previous co-expression analysis, and found that TF and its target gene identified by ChIP-seq have higher co-expression correlation than the control (*P* value < 2.2e−16). Using this TIP model, we produced a network with 272,627 edges and 20,179 nodes (~45% of the annotated genes and ~77% of the leaf expressed genes, Supplementary Data 10).

**Fig. 4:**
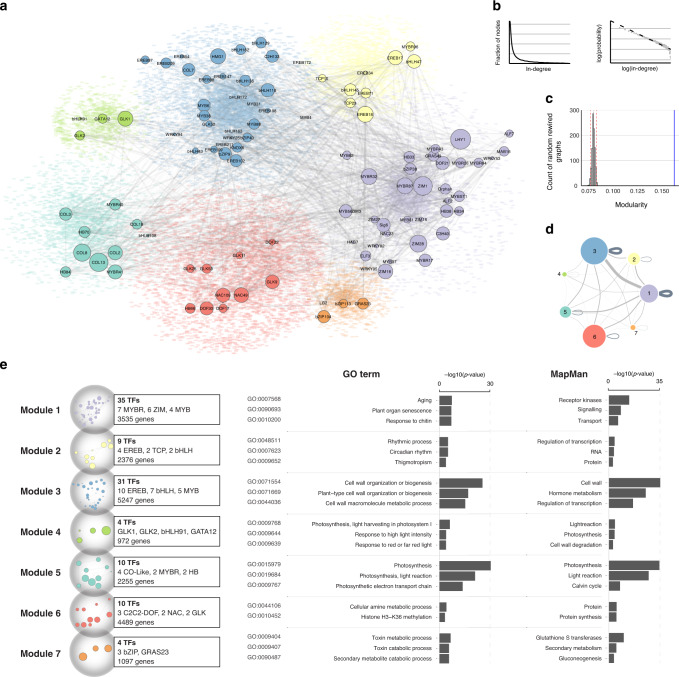
The maize transcription regulatory network exhibits properties of real-world networks. **a** Graph diagram showing the maize transcription regulatory network. The seven modules are labeled in different colors. TF nodes are shown as circles with size representing their expression level, and their target genes are shown as colored dots in the background. TF-to-TF edges are shown as gray lines between nodes. **b** Distribution of node in-degree values fits the power-law (dashed line). **c** Modularity of the network (blue line) is higher than the random control (histogram of maximum modularity values of 1000 rewired graphs). **d** Graph diagram showing TF-to-gene connections within and between modules. Each module is represented as a circle with size proportional to the number of nodes within. **e** GO-term and MapMan category enrichment analysis of genes in each module. Source data underlying Fig. 4e are provided as a Source Data file.

Real-world networks, such as the internet, social networks, and protein–protein interaction networks, frequently exhibit a scale-free topology with a power-law degree distribution^26^. In our maize transcription regulatory network, in-dgree of each node represents the number of TFs that could bind to, and potentially regulate this gene. We evaluated the in-degree distribution and found it followed a linear trend in the log-scale (*R*^2^ = 0.882, *P* value < 2.2e−16), as expected for power-law distribution (goodness of fit *P* value = 0.67), which is a landmark of scale-free networks (Fig. 4b).

In a scale-free network, nodes that appear more connected than others are called hubs, and they are critical for information flow^26^. We defined hub genes in our network as target nodes in the top percentile of the in-degree distribution (99th percentile, for a total of 206 genes). Interestingly, we found half of the hub genes were located nearby the TF highly occupied regions (Supplementary Data 7). Similar to those highly occupied regions, GO enrichment analysis showed that hub genes are enriched for regulatory functions, and many of them are TFs, consistent with the expected role for a hub node in the transcription regulatory network.

### Structural and functional modularity of the network

Biological networks often exhibit topological and/or functional modularity^27,28^. We tested for this predicted property by contrasting the maximum modularity in the network to a null distribution from an ensemble of random rewired graphs (H0: 1000 rewired graphs)^29^. The result confirmed that the network exhibits a significant increase in modularity (*P* value < 0.05, Fig. 4c). Next, we applied a partitioning algorithm (Gephi version 0.92) to determine relationships between subsets of network elements, and found that the network can be divided into seven modules (resolution 1.0). Each module contains ~27 to ~5% of the total nodes (Supplementary Data 11). These modules are not isolated and we found ~40% of the total edges occurring within each module, suggesting that TFs can regulate genes outside their own modules, and there are large information flows between modules (Fig. 4d).

Next, we test whether topological modularity could be related to function in known biological processes. We performed GO-term and MapMan functional category enrichment analyses for genes in each module, and found that they were indeed enriched for specific functions (Fig. 4e). For example, we found that module 4 (Fig. 4a, light green) is associated with photosynthesis related GO-terms, and it is enriched in targets of GLK1/GLK2, which are known regulators of photosynthesis^30^.

**Fig. 5:**
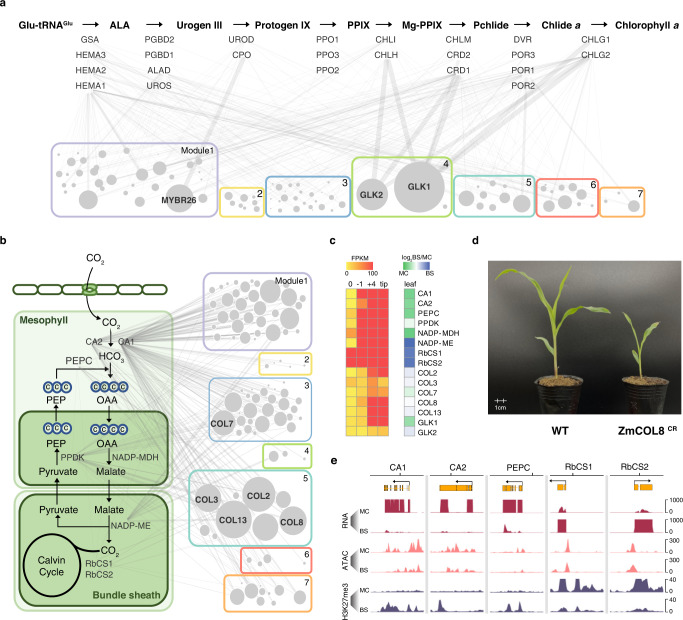
Identifying key regulators based on local TF connectivity. **a** The chlorophyll biosynthesis pathway. The genes encoding key enzymes for each step are shown below the arrow. The widths of the edge between TF and gene represent regulatory potential based on the TIP model. The 104 TF nodes are represented as circles and grouped into 7 modules, with size proportional to the sum of the regulatory potential. **b** The maize photosynthetic carbon pathway. Key genes encoding enzymes responsible for CO_2_ fixation and transport between mesophyll and bundle sheath cells are shown. **c** Heatmap showing the expression gradient of core C_4_ photosynthesis genes in four leaf sections from base to tip, as well as their mesophyll and bundle sheath cell-specific expression patterns. **d** Pale-green leaf and seedling lethal phenotypes of the COL8 CRISPR/Cas9 mutant. **e** Cell-type specific H3K27me3 in core C_4_ gene loci. MC mesophyll cells, BS bundle sheath cells. Source data underlying Fig. 5c are provided in a Source Data file.

However, each module contains thousands of genes of different functions and is too large to be assessed as a whole. We hypothesize that as the network can already provide clues to biological function at this scale, potential regulators of a smaller pathway might be identified based on connectivity at a local scale. We first tested this in the conserved chlorophyll biosynthesis pathway, which is known to be regulated by GLK TFs, as their mutations could disrupt the expression of photosynthesis genes^30,31^. To infer the contribution of each TF to a given pathway, we calculated the sum of the log transformed p-values that the ENCODE TIP probabilistic model generated for each TF-target interaction, based on binding intensity and proximity (Fig. 5a). We found that the top regulators of the chlorophyll biosynthesis pathway are indeed the two GLKs and an unknown MYBR26. Although the function of MYBR26 has not yet been studied in maize, its Arabidopsis homologs are involved in circadian regulation, further confirming our hypothesis^32^.

Next, we used this strategy to examine the maize C4 photosynthesis pathway, which lacks predefined regulators. It turns out that the top five TFs in connectivity ranking are CONSTANS-LIKE (COL) TFs (Fig. 5b, c). Previous studies of COLs in other plant species have shown that they play an important role in the regulation of flowering and photoperiod^33^. Since there are no maize COL mutants, we searched different maize CRISPR/Cas9 populations and found one line with a frame-shift deletion in the first exon of COL8 (Supplementary Fig. 4). The homozygous mutant has a pale-green and seedling lethality phenotype, supporting our hypothesis that the COL TFs are important for photosynthesis (Fig. 5d). Interestingly, for key C4 photosynthesis genes that are expressed specifically in either mesophyll or bundle sheath cells, we found that their gene loci are associated with cell-specific H3K27me3 marks, suggesting that they are regulated not just by a complex TF network, but also at the epigenome level (Fig. 5e).

### Conservation of TF-binding sequence preferences

**Fig. 6:**
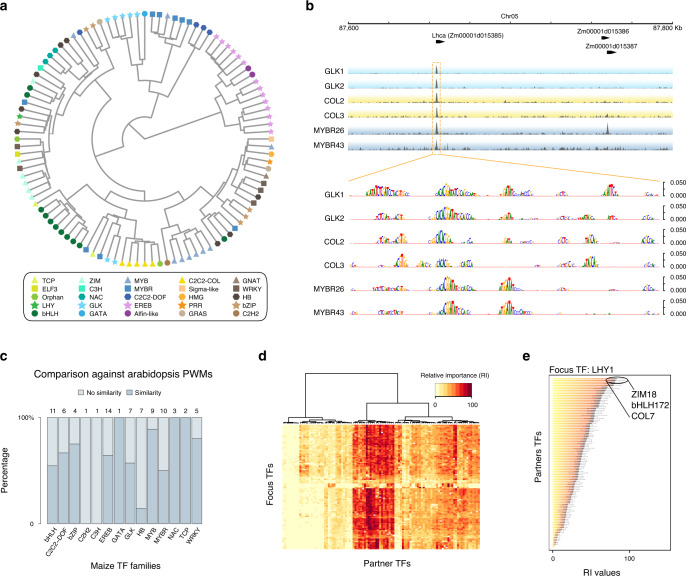
Machine-learning models of TF recognition sequence and co-binding. **a** TFs clustered by sequence binding similarity (predicted *k*-mer weights) derived from the sequence models. **b** Example browser tracks showing the promoter region of *Lhca*, which encodes the light-harvesting complex of photosystem I. Occlusion analysis for TFs targeting *Lhca* promoter showing scores of putative regulatory positions at base pair resolution derived from the models. **c** Conservation of recognition sequence between Arabidopsis and maize TF homologs. Numbers of TFs examined are shown at the top, and the TF family names are shown at the bottom. **d** Cluster of 104 TFs based on their relative importance value with co-binding TFs. **e** Top three co-binding partner TFs of LHY1 based on RI ranking. Source data underlying Fig. 6c, d are provided as a Source Data file.

To model TF binding from sequence, we applied a bag-of-*k*-mers machine-learning model^34^ to discriminate TF-binding regions from other regions in the genome, which resulted in reliable models for all the TFs (fivefold cross-validation, average accuracy for each TF > 70%) (Fig. 6 and Supplementary Datas 12 and 13). Using average *k*-mer weights from the models, we derived a distance matrix among TFs, and summarized TFs relationships (Supplementary Data 13). After removal of singleton families, we observed that for 85% of the TFs families, most of their members (≥50%) cluster into the same group in a dendrogram (Fig. 6a and Supplementary Fig. 5).

This observation prompted us to evaluate whether TF sequence preferences have persisted across angiosperm evolution, as TF protein families are frequently well conserved in plants. Using the top 1% of the predictive *k*-mers for each TF, we examined their similarity to a large collection of Arabidopsis TF in vitro binding position weight matrices (PWMs)^35^. After removal of families that did not have a counterpart (or were poorly represented), we found that 50 out of 81 (61%) of the evaluated TFs preferentially matched PWMs to their corresponding family in Arabidopsis (*P* value < 0.001, Fig. 6c). Overall, our finding suggests a strong constraint over more than 150 million years, which also agrees with the reduced SNP variation at the TF-binding loci (Fig. 3a).

### TF co-binding is context specific

Any given TF with an average recognition site of 6.8 bp could have affinity for roughly a third of a million locations across the maize genome^34,35^, while on average, they could only bind to 20,000 sites. In addition, members in the same TF family could target different genes despite having similar sequence preferences. Hence, achieving the observed TF binding specificity would likely require extra cues. It has been shown for human TFs that co-binding and combinatorial recognition of *cis*-elements are key for specificity^5^. To test this, we created machine-learning models based on co-localization information to learn nonlinear dependencies among TFs using the ENCODE pipeline^5^. To fit a model for each TF (i.e., focus TF or context), we built a co-localization matrix, by overlapping peaks for the focus-TF with peaks of all remaining TFs (i.e., partner TF). The co-localization model was aimed to discriminate between the true co-localization matrix and a randomized version of the same^36^. The output of each model is a set of combinatorial rules that can predict TF binding. For each TF, the average of 10 models with independent randomized matrices have an area under the receiver operating curve >0.9 (Supplementary Data 14). This high performance supports the hypothesis that TF co-localization has vast information content to determine binding specificity in the maize genome.

Using the rules derived from the co-localization models, we scored the relative importance (RI) of each partner TF for the joint distribution of the set of peaks for a given context (Fig. 6d). In this way, the co-localization models allow us to examine the importance of a partner TF to be predictive (in a quantitative fashion) of the binding of a context, and not from simple co-occupancy. To obtain a global view from the model results, we calculated the average RI of a TF across all focus TFs. We observed that the whole set shows a trend toward medium-to-low average RI values (i.e., ≤60 RI, more context specific), with fewer TFs being predictive for a large number of focus TFs (i.e., >60 RI, high-combinatorial potential, Supplementary Fig. 6). For example, among the 104 TFs, LATE ELONGATED HYPOCOTYL (LHY) is the most highly expressed one in the differentiating leaf section^10^. LHY encodes a MYB TF that is a central oscillator in the plant circadian clock^37^, and the top three predicted partner TFs based on RI are ZIM18, bHLH172, and COL7 (Fig. 6e). Although their functions have yet to be characterized in maize, their Arabidopsis homologs are involved in jasmonic acid signaling, iron homeostasis, and flowering time regulation, respectively, all of which are tightly coupled to the circadian clock^38^.

Clustering according to the RI values revealed a group of TFs that have large RI across many focus TFs (Fig. 6d). The large RI score means that a TF is highly predictive of the binding in a specific context. Interestingly, some of them are homologs to Arabidopsis TFs involved in hormone signaling (e.g., ZIM TFs in JA signaling). Some belong to families that are known for protein–protein interactions, such as the MYB-bHLHs and the ZIM-MYB-bHLHs TFs^39–41^, suggesting that these high-RI factors could function to integrate multiple inputs to coordinate transcriptional output.

In summary, our finding confirms that co-binding could be the key to explain how TFs with similar sequence preferences could target different genes and control different biological functions. The co-localization model also revealed a large combinatorial space for TF-binding sites that likely favors the occurrence of specific combinations, which could facilitate rapid diversification of the regulatory network during speciation.

### Conservation of the TF regulatory interactions among grasses

**Fig. 7:**
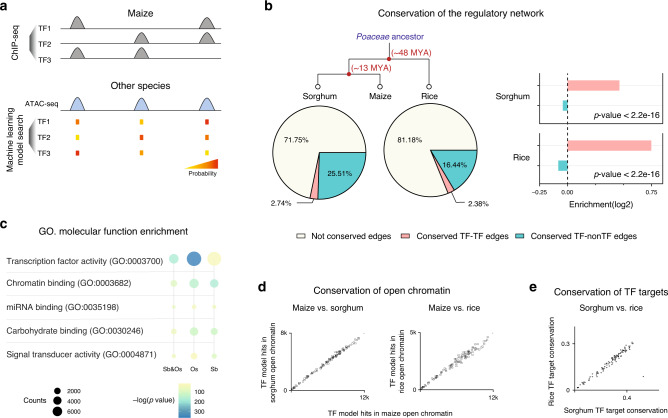
Conservation of the TF regulatory interactions. **a** Schematic diagram showing how the TF binding *k*-mer model is used to search the open chromatin regions of maize synteny gene in other species, to infer conservation of the regulatory interaction. **b** Pie chart showing percentage of conserved edges in sorghum (left) and rice (right), and bar plot showing TF-to-TF edges are more conserved than TF-to-non-TF edges (*P* value; two-sided Fisher exact). **c** Diagram showing enriched GO-terms for conserved maize TF-target genes. From left to right: conserved in both sorghum and rice, conserved in rice and conserved in sorghum. The size of the circle indicates the number of genes, and the color is scaled to −log10(*P* val) (two-sided Fisher exact). **d** Number of recognition sequences of each TF predicted in maize, sorghum, and rice open chromatin. **e** The axis indicates the percentage of target conservation of each maize TF in rice and sorghum, inferred from the TF model matches in their synteny target gene open chromatin.

Next, we used the machine-learning models to investigate how the transcription regulatory network evolved in grasses. To do so we performed ATAC-seq in sorghum and rice, and obtained open chromatin sequences of their syntenic maize genes. We then inferred network edge conservation based on whether the model of maize TFs could predict binding in the open chromatin of the synteny target genes in sorghum and rice (Fig. 7a). For example, we found predicted TF-binding events in 68% of the syntenic open chromatin regions in sorghum. Looking at the predicted network edges from syntenic TF to syntenic genes, we inferred that ~28% of the edges in the maize network were conserved in sorghum, and ~19% were conserved in rice (Fig. 7b).

Comparative studies of animal regulatory networks have shown that the core network, which consists of TF-to-TF connections, are frequently more conserved than the rest^42^. If the same is true in plants, we would expect TF genes to be enriched in the set of conserved targets. Hence, we conducted GO enrichment analysis and the results agreed with our expectation, with target nodes enriched for transcription regulatory roles (Fig. 7c). In addition, we also examined the ratio of conserved edges, and found that the TF-to-TF edges were over-represented compared to TF-to-non-TF edges in both sorghum and rice (Fig. 7b).

Data from modENCODE revealed a strong correlation between the human and mouse homologous TF recognition sites, and the prevalence of TF recognition sites in their open chromatin is under selection^43^. To test these in plants, we calculated the number of matches of each TF model in the open chromatin regions of maize, sorghum, and rice, and found that they are indeed correlated (Fig. 7d). In addition, for each maize TF, the numbers of conserved targets found in rice and sorghum are also correlated (Fig. 7e), suggesting similar selection pressure during plant and animal evolution.

Taken together, our findings suggest that plants and animals might have adopted a similar strategy to evolve transcription regulatory network, and the rewiring of the network occurs in a hierarchical fashion, with the core network nodes at the top of the transmission of information being more conserved than those at the bottom.

## Discussion

Previous studies on plant transcription regulation have often been limited to individual TFs or a small number of TFs that have been previously linked to the biological process in question. These approaches could underestimate true regulators that act in a redundant or additive manner, and often result in an oversimplified linear or hierarchical regulatory graph. To overcome this, we attempted to reconstruct the whole transcription regulatory network without bias, by using large-scale ChIP-seq to systematically profile TFs binding (Fig. 1). By doing so, we obtained a network that covers over 77% of the leaf genes, with 272,627 edges and 20,179 nodes, which means a gene could receive redundant regulatory inputs from dozens of TFs. Although it might be new to plants, such regulatory complexity and redundancy are well recognized in animal models^3–6^, and these could be of evolutionary advantage, such as resistance to random perturbations, and serving as a reservoir of stored neutral mutations for future usage. In order to understand complex and redundant systems, a bottom-up study toward a systems biology approach is most suited, and we need to study the transcription regulatory network as a whole, not just the individual components.

One interesting feature of the maize transcription regulatory network is its scale-free topology (Fig. 4). It lacks a single characteristic node in-degree, and is dominated by a small number of highly connected hubs. A key advantage of the scale-free topology is the increased tolerance to random failures^26^. However, the hubs could become the vulnerabilities, if they are not redundant. Using chlorophyll biosynthesis and C4 photosynthesis as examples (Fig. 5), we showed that mutations of highly connected TFs could disrupt plant growth and development. More importantly, it demonstrated how network connectivity data could be used to predict biological functions and identify potential regulators. In addition, the observed structural and functional split of the network indicates how complicated gene expression is fine tuned. For example, the presence of multiple TFs across modules to regulate genes in the same pathway suggests intricate modes of actions to coordinate transcription, in contrast to the classic view of one single or few master regulators controlling a biological process or pathway.

Despite more than one billion years of evolution and lack of detectable sequence conservation, we found that the plant and animal regulatory codes display striking similarity^2,5^. For example, we found that maize TF co-binding could be key for specificity, and it is considered as the most important mechanism of gene regulation in mammalian cells. Co-binding could be beneficial for rapid diversification of the regulatory code. This is because, from an information standpoint, it enables a large variety of transcriptional outputs using synergistic and combinatorial inputs of a small number of TFs. For instance, taking sets of five distinct TFs (i.e., the mode of distinct TF-binding sites in maize open chromatin regions) from the 104 TF repertoire could generate millions of possible combinations. In addition, high-combinatorial factors, such as MAX and P300, play key roles in transcription regulation in mammalian cells^43,44^, while those in plant have yet been characterized in detailed, and deserve further attention.

Most of the important crop genomes have now been sequenced, and numerous GWAS studies have been carried out to associate agricultural traits to QTLs. However, functional interpretation of the genome noncoding regions remains a challenge. In this study, we generated extensive and non-biased TF-binding data that could connect sequence variations in QTLs to trans-regulators, to highlight the molecular mechanism underlying complex phenotypes, and should greatly facilitate future functional genomic analysis. Without knowing the precise location and function of these *cis*-regulatory elements in the open chromatin regions, crop genome editing often randomly targets the 5′ upstream region of genes to generate expression variations, which is rather like looking for a needle in a haystack. With the extensive TF-binding information, re-sequencing data, and TF-binding and co-binding machine-learning models, we could now begin to establish pipelines to predict effects of noncoding variants, both common and rare, on TF binding, to pinpoint causal sites. Not to mention the possibility of being able to predict and generate novel variations not seen in nature could fundamentally change future plant breeding.

## Methods

### Plant materials and growth conditions

Seeds of maize (*Zea mays* B73), sorghum (*Sorghum bicolor* BTx623), and rice (*Oryza sativa* Nipponbare) were germinated on wet paper in petri dish at 28 °C for 2 days. On the third day, plants were transferred to compose with vermiculite in a plant growth incubator under the conditions of 12:12 Light/Dark, 30 °C Light/22 °C Dark and 70% relative humidity. Nine-day-old seedlings of maize and sorghum, and 14-day-old rice seedlings were used for the experiments.

### ChIP-seq using maize leaf protoplast

We have developed an efficient protoplast isolation and transformation method to express maize TF constructs for ChIP-seq (Supplementary Method 1). Short videos showing the critical steps can be viewed online (https://space.bilibili.com/511150037/).

### Preparation of hyper-stable Tn5

Transposase Tn5 tagmentation is one of the most convenient library preparation methods available. However, homebrew Tn5 tends to lose activity rapidly due to protein aggregation. To improve the reproducibility, we fused the Tn5 to the C-terminal of the *E. coli* elongation factor Ts, which is often used as N-terminal tag in the purification of aggregation prone proteins^45^. This hyper-stable TS–Tn5 transposase enabled us to improve the consistency and reduce the cost of ChIP-seq library construction (Supplementary Method 1). All plasmids could be obtained from Addgene (accession #127916).

### RNA-seq, histone ChIP-seq, and ATAC-seq

Illumina TruSeq RNA sequencing libraries, tagmentation-based H3K27me3 ChIP-seq libraries and ATAC-seq libraries were constructed using maize, sorghum, and rice seedling tissues as previously described^46,47^ (Supplementary Methods 2–4).

### Alignment of ChIP-seq and ATAC-seq reads

Reads were mapped to the unmasked maize genome (B73 RefGen_v4) using Bowtie 2 (version 2.2.5) under the default parameters with −3 100 trimming option. Next, unmapped reads were filtered using SAMTools view (version 1.3) with options -F 4 and -q 10, and duplicated reads were removed using SAMTools rmdup.

### Quality control for TF ChIP-seq

To assess the reproducibility between biological replicates, we used deepTools (version 3.2.0) multiBamSummary to calculate their Pearson correlation. Biological replicates with Pearson correlation coefficient ≥ 0.8 were retained for further analyses. Next, we used the PhantomPeakQualTools (version 1.14) to assess the signal-to-noise ratios based on normalized strand cross-correlation coefficient (NSC ≥ 1.05) and relative strand cross-correlation coefficient (RSC ≥ 0.8) of the individual alignment files. Pairwise replicates that passed the quality control were used to call peaks with the SPP peak caller using relaxed parameters (-npeak = 300,000), with corresponding input as control. Before reproducible peak calling, we subtracted a list of genome blacklist regions, together with peaks located to mitochondria/chloroplast produced from SPP using the BEDTools (version 2.27.1). It should be noted that the TF construct in the form of plasmid DNA was transformed into protoplasts. Reads originated from the plasmid could also be detected in the ChIP-seq library, and part of them could be mapped to the exons of the TF gene itself. Hence, for each TF ChIP-seq, we subtracted the peaks located in its own gene locus using BEDTools. Reproducible peak calls were obtained with the IDR statistical framework (version 2.0.3), as implemented in the PhantomPeakQualTools package, using 1% IDR as the threshold. The BigWig and peak files generated have been deposited in NCBI GEO (GSE137972) and could also be viewed online at www.epigenome.cuhk.edu.hk/C3C4.html.

### Analysis of TF peaks and open chromatin

For each TF, we obtained a set of reproducible peaks. Peaks were merged based on summit positions at around twice the distance of a nucleosome (300 bp) with BEDTools merge -d 300 for a total of 144,890 TF-binding loci located in chromosomes 1 to 10 (Supplementary Data 5). To assess the statistical relation between a TF-binding loci and open chromatin regions we used a permutation test (a randomization-based approach) that is implicitly considered genome complexity, as implemented in the regioneR package (version 1.14.0). The association between open chromatin regions (ATAC-seq peaks) and TF-binding loci was tested using a per chromosome randomization of TF-binding loci (the number of overlaps as the evaluation function and 10,000 permutations). The observed value appears far from the limit of the threshold (*P* value < 0.01) of the random distribution.

### Sequence conservation in TF-binding regions

We used the SNPs from the maize haplotype (HapMap version 3.2.1)^19^ to calculate the SNP density over 100 bp (nonoverlapping) windows through VCFtools (version 0.1.17) using the option SNP density 100. Next, we calculate the distances between the midpoint of each SNP density bin and the TF peak summits, to a maximum considered distance of 1 kb. The mean SNP density was calculated at each distance value, and a sliding window of 100 positions was used to average the mean SNP count to plot a smooth line.

### TF binding and eQTL

We obtained the maize eQTLs from a previous study^21^, to test whether there is significant relationship between TF-binding region (all TF-binding loci and independently for each TF) and nucleotides identified as genetic regulators of gene expression acting in *cis* (i.e., eQTLs around 1 Mb of the gene with which was associated). First, we used the union set (individual occurrences) of eQTLs in foliar tissue, with higher effect and lowest *P* value for each gene in the maize genome across leaf tissues. The set of all SNPs, were obtained from the maize HapMap 3.2.1 for all taxa in the RNA-seq, using a minimum read count = 5 (the same filtering criteria applied in order to run the eQTL analysis). We quantified the enrichment of best *cis*-eQTL hits (relative to all SNPs) within TF-binding loci vs. control regions. Control regions are located ≥5 kb from the nearest TF-binding region, with half the size of the respective TF-binding region flanking each side. In order to control for the possible confounding effects of distance to the nearest gene, we run the analysis separately for regions proximal (<2 kb) and distal (>2 kb) from genes (Supplementary Data 8). Finally, we plot the posterior distribution of *cis*-eQTLs SNP frequency, relative to all SNPs, using a beta-binomial distribution with a Beta (1,1) prior. To calculate the enrichment of eQTLs on TF-binding regions vs. control regions, we found the ratio between the two beta-binomial distributions and determine the 95% confidence interval, as the percentile 2.5 and percentile 97.5. The same analysis was repeated for Exons, Introns, 5′ UTR and 3′ UTR to provide a contrast for TF-binding regions.

### TF binding and GWAS hits

To test for a significant relationship between TF-binding region (all TF-binding loci and independently for each TF) and quantitative traits, we quantified the identified SNPs for an assortment of traits measured in the US NAM population. First, we run GWAS for traits that represent the diverse levels of complexities, and which could have been influenced by the regulatory action of the measured TFs. The phenotypic data we used, includes metabolites^48^, leaf architecture^23^, and photoperiodicity^24^. Variants (SNPs) considered for input correspond to imputed NAM SNPs (HapMap 3.2.1) with MAF ≥ 0.025. Following the construction of a kinship matrix, we used Gemma (v0.98.1) to fit a linear mixed model –lmm 1, and flowering time (days after anthesis) as a covariate for metabolites and leaf architecture. After running Gemma, we considered GWAS hits, as those variants with significant association, after applying a high-confidence threshold (false-discovery rate (FDR) 5%). We quantified the enrichment of GWAS hits relative to all SNPs (within TF-binding region vs. control regions). Control regions are located ≥5 kb from the nearest TF-binding region, with half the size of the respective TF-binding region flanking each side. In order to control for the possible confounding effects of distance to the nearest gene, we rerun the analysis separately for regions proximal (<2 kb) and distal (>2 kb) from genes (Supplementary Data 9).

### Inference of a transcription regulatory network

To identify the target genes of each TF, we used a probabilistic model called TIP^25^, designed to quantitatively measuring the regulatory relationships between TFs and genes. Briefly, for each TF, TIP builds a characteristic profile of binding surrounding the start coordinate of genes. Next, the binding profile is used to weigh the TF binding (peaks) associated with a given gene. To identify the most confident targets of a TF, we employed a stringent threshold with a false-discovery rate of 5% (FDR = 0.05). We build a directed graph (i.e., the 104 TFs as source nodes target genes as target nodes) from the interactions passing the threshold and used the −log10(*P* value) as weight (Supplementary Data 10).

### TF network analyses

To determine if the node in-degree follows a power-law degree distribution (i.e., scale-free networks) the in-degree distribution was converted to a QQ-plot, and fit to a linear regression (*R*^2^ = 0.882, *P* value < 2e−16), which is paramount of a power-law distribution^49^. To confirm our result, we further investigated the goodness of fit with bootstrapping (no_of_sims = 1000) using the poweRlaw R package (version 0.70.2). The goodness of fit (i.e., Kolmogorov–Smirnov test) analysis resulted in a *P* value of 0.692, which does not allow the rejection of the null hypothesis that claims that the distribution is a power-law.

Gene Ontology term analysis was performed in a set of candidate target hubs at the extreme of the in-degree distribution (i.e., nodes in the 99th percentile). PLAZA (v4.0 Monocots) was used for GO annotations and GO enrichment analysis. All the genes in our graph were supplied as a background.

To test for topological modularity, we built a null distribution from an ensemble of random rewired graphs (H0: 1000 rewired graphs), while maintaining the number of nodes and number of edges per node, calculating for each a maximum modularity parameter. This analysis shows statistically significant differences in modularity, which was large in our graph (*P* value < 0.05) vs. the null distribution. It should be noted that current algorithms for the estimation of modularity have a resolution limit that can fail to detect small communities. To explore the number of modules in the filtered graph, we determined modularity across several resolutions using Gephi (version 0.9.2) with resolution limits between 0.7 and 1.2 (Supplementary Data 11). At resolution 1.2 one single module includes 64% of the TFs, and at 1.1 one single module includes 45% of them. We decided to explore modules at resolution equal or less than 1.0, as any single module include more than 30% of the TFs, for further analysis. We tested if the structural modularity was related to a functional modularity by evaluating each module for enrichment in GO terms and MapMan functional categories (Fig. 4e).

### TF recognition site sequence model

To study the sequence preferences of each TF, we fit a bag-of-*k*-mers model to discriminate between TF-binding regions, from sequence with similar GC% content^34^. In brief, each set of peaks and its control regions correspond to a collection of individual sequences, labeled with a list of sequence labels (i.e., *y*) (1 for peaks and 0 for control regions). Next, the sequences are represented as a *x* matrix of tokens corresponding to collapsed *k*-mers (*k* = 7) filled with a weighted version of the token frequencies. The tokens weights correspond to the TF*IDF, or the product of the token frequency in each sequence, and its inverse collection frequency. The bag-of-*k*-mers model results from fitting a regression curve, *y* = *f*(*x*) (i.e., a regularized logistic regression). Logistic regression as used with the bag-of-*k*-mers correspond to the implementation of the python library scikit-learn (version 0.19.0).

We fit a model for each of the TFs (fivefolds cross-validation), and averaged the obtained *k*-mer weights obtained from the five models (Supplementary Data 12). The averaged weights of the scored *k*-mer vocabularies were further used to systematically contrast the binding profiles between TF. First we calculated the ‘spearman’ correlation between TFs, and used as input for the “hclust” function from the fastcluster R package (version 1.1.25) with method “ward.D”. We used a dynamic tree cut method to the result form the “hclust” function we identified 10 clusters, in which most of the TFs clusters with members of the family to which belong (Supplementary Data 13).

### Occlusion and saturation mutagenesis maps of TF-binding regions

To reveal regions that are responsible for the prediction of a “bag-of-*k*-mers” model we calculated importance scores for each of the nucleotides in a given sequence. First, we calculated nucleotide occlusion score from subtracting the probability obtained for label 1 with a sequence of interest, and the probability obtained when a given position is replaced by an *N* in the same sequence of interest. This method decreases the frequency of the *k*-mer that includes the given position without any other change in the input matrix. The rationale behind this analysis is that positions that are important for the TF binding should decrease the probability scored by the model. Second, we calculated nucleotide mutagenesis scores from subtracting the probability obtained for label 1 with a sequence of interest (original score), and the probability when a given position is replaced by each of other nucleotide in the same sequence of interest (i.e., change A by C, G, or T). Using this method, the frequency of the *k*-mers that includes the given position is replaced with counts for different *k*-mers. The use of nucleotide occlusion score and nucleotide mutagenesis scores provide a fine resolution to determine the potential of a mutation to affect the TF binding, which could guide future experiments and variant interpretation (Supplementary Fig. 5).

### Comparison with Arabidopsis TF

To determine the similarity in sequence binding preferences between maize TFs and Arabidopsis TFs, we contrasted the top 1% *k*-mers to the collection of DAP-seq PWMs using TOMTOM from the MEME suite (version 5.0.5) and a collection of TF-binding PWMs derived from in vitro binding profiles of a large number of Arabidopsis TFs^35^. To a given maize TF we determined the most similar Arabidopsis TF (PWM) the one to which the largest number of *k*-mers hit (Fig. 6c). We considered a hit between a *k*-mer and a PWM as positive with a *P* value of less than 1e−4, and a PWM scores greater than 13.28 bits. This parameter has been defined as a gold-standard to determine positive PWMs hits previously^50^.

### TF co-localization model

We implemented the co-localization model that has been used to study the combinatorial binding of human TFs, in the context of the binding preferences of specific TFs^5^. In brief, we fit machine-learning models to capture quantitative dependencies between TF-binding profiles, across different genomic contexts. Hereafter, genomic context can be defined as a collection of genomic locations or intervals that correspond to a common annotation. For our purposes each context corresponds to genomic locations of peaks for a particular TF (focus TF). For each TF, we computed normalized ranks to represent each binding event with a value between 0 and 1. The normalized rank of a peak is given by (*R* − *r*)/(*R* − 1); where *r* is the rank of the set of *R* peaks when sorted numerically by signal value. The absence of overlap between a partner TF and the focus TFs correspond to 0. After rank normalization, we overlapped the peaks of a focus TF with the summit of the peaks of all partners TFs (all the other TFs) to obtain a co-localization matrix for the focus TF. The co-localization matrix was randomized (*n* = 10 times), to generate negative sets that served as input for a discriminative machine-learning algorithm RuleFit3^36^. The overall goal of the models is to capture quantitative dependencies between TF-binding profiles that are enriched in the true co-localization matrix vs. the randomized version.

### TF co-localization rule learning

We used the RuleFit3 algorithm to learn TF co-localization rules. First, a random forest learns ensembles of decision trees that discriminate the true co-localization matrix from the randomized version. The learned decision trees are them used as a repository of rules with discriminative power. Such repository constitutes a smaller space of combinatorial rules than all the possible combinations of partner TFs. Second, the algorithm uses the repository of rules as inputs to learn a final nonredundant set of discriminative rules. A typical rule can consist in one, or a combination of TFs, for instance, TFA, TFB, and TFC, could be quantitatively associated in the following rule (0.4 < TFA ≤ 1) AND (0.6 < TFB ≤ 1) AND (0.3 ≤ TFC ≤ 1), to explain the binding profile of TFD (focus TF). The set of final rules represent combinations of partner TFs with nonrandom relationships between them, within a single genomic context (i.e., enriched in the true co-localization matrix compared to the randomized version). To ensure the robustness of our approach, we evaluated the discriminative power of the learned models by training several models for each focus TF, contrasting the true co-localization matrix to multiple randomized (*n* = 10) control versions. To obtain robust metrics, we obtained averages and medians of accuracy, and other model-based scores over the multiple models.

### Relative and differential importance scores

In brief, the co-localization model allows us to compute a RI score (i.e., the RI, their relative contribution to the discriminative performance of the model) of each partner TF in a single genomic context. The RI score of a TF ranges from 0 to 100, and each RI value represents a partner TF-focus-TF relationship that mathematically corresponds to the relative contribution of a TF to the performance of a model trained to distinguish the joint distribution of all binding profiles in a single genomic context from an equivalent randomized control^5^. Intuitively, we can expect that TFs that co-localize with several other TFs across a single genomic context (i.e., a significant fraction of the peaks of a focus TF), will have a high RI in that context. The summary of all the models is a matrix, in which each context is represented as a row, and each cell contains the RI values of each TF organized in columns. We clustered independently rows and columns (hierarchical clustering, Euclidean distance, and Ward linkage) to show similar RI pattern across similar contexts (column clustering), and similar dependencies of partner TFs (row clustering) (Supplementary Fig. 6a). The averaged RI values, across the columns of the matrix, can be seen as the overall combinatorial potential of each TF. The distribution of the averaged RI values identified a few TFs that are important across many contexts (i.e., average RI > 60). However, we found that more frequently, TFs are only important for a subset of contexts (Supplementary Fig. 6b). We derived a differential importance (DI) score for each TF, that describe how the RI changes between subsets of peaks (i.e., genic proximal and distal), and calculated the average DI to score genome-wide bias. The average DI score can be seen as the overall localization bias, with some TFs preferentially important to predict in the proximal contexts, while others are important in the distal context. The stack of DIs for all focus TFs (Supplementary Fig. 7a) as well as the distribution of the average DI (Supplementary Fig. 7b) show TFs with differences in the RI between proximal and distal regions. It should be noted that the co-localization model is a multivariate analysis which have to be fit each time that a new TF binding profile is available. As our data represent a sample of all the TFs present at a given time in the tissue, the RI of a TF in a context can change on the light of new information.

### Infer regulatory interaction using co-expression

To construct a gene regulatory network based on published maize RNA-seq data^51–53^, we used GEne Network Inference with Ensemble of trees (GENIE3) to predict gene regulatory relationships^54^. Next, we extracted the GENEI3 co-expression weight of the 104 TFs to their target genes, and compared to those of TF to non-target genes.

### Reporting summary

Further information on research design is available in the Nature Research Reporting Summary linked to this article.

## Supplementary information

Supplementary Information

Peer Review File

Reporting Summary

Description of Additional Supplementary Files

Supplementary Dataset 1–14

## Data Availability

Data supporting the findings of this work are available within the paper and its Supplementary Information files. A reporting summary for this article is available as a Supplementary Information file. The datasets generated and analyzed during the current study are available from the corresponding author upon request. Sequencing data that support the finding of this study have been deposited in the NCBI SRA database under the accession number PRJNA518749. Processed data have been deposited in the NCBI GEO database under the accession number GSE137972. Source data are provided with this paper.

## Source data

Source Data

## Code availability

Code to reproduce analyses is available at https://bitbucket.org/bucklerlab/p_transcriptionfactors/src/master/.
