# Author Correction: Reconstructing the maize leaf regulatory network using ChIP-seq data of 104 transcription factors

**DOI:** 10.1038/s41467-023-37423-x

**Published:** 2023-03-22

**Authors:** Xiaoyu Tu, María Katherine Mejía-Guerra, Jose A. Valdes Franco, David Tzeng, Po-Yu Chu, Wei Shen, Yingying Wei, Xiuru Dai, Pinghua Li, Edward S. Buckler, Silin Zhong

**Affiliations:** 1 The State Key Laboratory of Crop Biology, College of Agronomy, Shandong Agricultural University, Shandong, China; 2 State Key Laboratory of Agrobiotechnology, School of Life Sciences, The Chinese University of Hong Kong, Hong Kong, China; 3 Institute for Genomic Diversity, Cornell University, Ithaca, NY USA; 4 Department of Statistics, The Chinese University of Hong Kong, Hong Kong, China; 5 School of Integrative Plant Sciences, Section of Plant Breeding and Genetics, Cornell University, Ithaca, NY USA; 6 Agricultural Research Service, United States Department of Agriculture, Ithaca, NY USA

**Keywords:** Transcriptomics, Gene regulatory networks, Gene expression, Plant molecular biology

Correction to: *Nature Communications* 10.1038/s41467-020-18832-8, published online 09 October 2020

The original version of this Article contained an error in Fig. 5e, in which the labels of the cell-types MC and BS were swapped for all three sets of data, RNA, ATAC, and H3K27Me3. This has been corrected in both the PDF and HTML versions of the Article.

