# Supplementary material for: Reconstructing the maize leaf regulatory network using ChIP-seq data of 104 transcription factors
Source: Nat Commun. 2020 Oct 9;11:5089. doi: 10.1038/s41467-020-18832-8 (PMC7547689; doi:10.1038/s41467-020-18832-8)
Supplement: Supplementary file 1 — Supplementary Information [file 41467_2020_18832_MOESM1_ESM.pdf]

Reconstructing the maize leaf regulatory network using ChIP-seq data of 104 transcription factors

Tu *et al.*

Supplementary Method 1. TF ChIP-seq protocol

Preparation of hyper-stable Tn5 transposase

HS-Tn5 transposon construct

The Tn5-intein-CBD was PCR amplified from pTXB1-Tn5 (Addgene plasmid #60240) and the full-length elongation factor Ts was PCR amplified from E. coli DNA. The two fragments were then cloned into pET-30a using Gibson assembly, and the plasmid generated has been deposited to Addgene (Addgene plasmid #127916).

Purification of HS-Tn5

1) To induce the HS-Tn5, the pET30-HS-Tn5 was first transformed to Rosetta (DE3) cells, and a single colony was picked and grown overnight in 10 mL LB (supplemented with 1% glucose, 50 ug/mL kanamycin and 20 µg/mL chloramphenicol) at 37°C. In the following day, 1 mL of the overnight culture was used to inoculate 200 mL LB without glucose until the OD600 reached 0.4~0.6 in a 37 °C shaking incubator. The cells were then transferred to 20°C shaking incubator, and IPTG was added to 0.1 mM to induce the HS-Tn5 expression. After 3-5 hours induction, cells were spun down, washed with PBS supplemented with 1 X PMSF and 10% glycerol, and the pellets were stored in aliquot at -80°C freezer. Around 0.5 mL of HS-Tn5 at 1 OD280 (11 uM) could be purified from one pellet originated from 100 mL culture.

2) To extract the HS-Tn5, one pellet (from 100 mL culture) was resuspended in ~3 mL cold Tn5 Lysis Buffer:
   10 mM NaCl
   20 mM Tris-HCl pH 8.5
   1 mM EDTA
   10% glycerol
   0.1% (w/v) Triton X-100
   0.1% (w/v) NP-40
   0.1% (w/v) DOC
   1 X protease inhibitor
   20 µg lysozyme

3) After around 10 min incubation with occasional vortex on ice, the cleared lysate was transferred to 6 eppendorf tubes and centrifuged at max speed for 10 min at 4°C to remove the debris. If the lysate was too viscous to be spun down, we often sonicated the tubes in Bioruptor (1-2 min 30/30 ON/OFF cycle, low power) to shear the bacteria genomic DNA. NaCl (4 M) and 10% polyethyleneimine were added to a final concentration of 0.8M and 0.25%, respectively. The lysate was then centrifuged twice at max speed for 20 min at 4°C to remove the genomic DNA.

4) Pack a gravity flow column with 1 mL chitin resin (NEB) and washed with 10 mL of Column Buffer:
   0.8 M NaCl
   20 M Tris-HCl pH 8.5
   0.1 mM EDTA
   10% glycerol
   0.1% (w/v) Triton X-100
   1 X PMSF

5) The HS-Tn5 lysate was loaded onto the column after passing through a 0.6 μm nylon filter to remove debris that could block the column. The first flowthrough was reapplied to the column. The column was then washed with 10 mL of Column Buffer, 2 mL of Column Buffer supplemented with 2 M urea, and finally another 10 mL of Column Buffer. To cleave the intein between HS-Tn5 and the chitin binding domain, the column was flushed with 1 mL of cold Column Buffer with 50 mM DTT. The column was then sealed; the resin is covered with 200 μL Column Buffer with DTT, and stored in the fridge for at least 36 hours.

6) After DTT cleavage, 2 x 2 mL of Elution Buffer was added to elute the HS-Tn5. To remove the DTT, the combined elute was loaded on an ultrafiltration spin column (Pierce Protein Concentrator PES, 30K MWCO, 2-6 mL), washed twice and finally concentrate to <200 μL in elution buffer:
   0.5 M NaCl
   10 mM Tris-HCl pH 8.0
   0.1 mM EDTA

7) To further remove the small molecular weight proteins, the concentrated HS-Tn5 was spin through a Zeba™ Spin Desalting Columns (40K MWCO, 0.5 mL) equilibrated with the Elution Buffer. The concentration of HS-Tn5 was then determined by OD280 measurement (11 uM ~ 1 OD280). For long-term storage at -20°C, equal volume of glycerol was added to the Tn5 solution, and β-mercaptoethanol (14.3 M) or DTT (100 mM) was also added to 1 mM. The unassembled HS-Tn5 can be stored at -20°C.

Loading adapter to HS-Tn5

1) The two Tn5/Nextera adapters were prepared using 100 μM oligo stock solutions (Tn5-ME-A, Tn5-ME-B, and Tn5-ME-Rev). Equal volumes of the adapter oligo solutions were mixed, heated to 95°C in a PCR machine, and slowly chilled to 20°C. Equal volume of the two adapters were then mixed to form the 50 μM A/B adapter stock. To assemble the transposome (*i.e.*, the stable complex between HS-Tn5 plus the transposon DNA), equal molar of Tn5 and A/B adapter were incubated at 25°C for 1 hour. Glycerol can be added to 40-50%, and the assembled Tn5 can be stored at -20 °C for a few weeks. The activity of the assembled transposome was tested by incubating 100 ng of lambda DNA with 1 μL transposome (~11 uM) in 20 μL Tagmentation Buffer:
   20 mM Tris-HCl buffer pH 8.0
   10 mM $MgCl_2$
   15% DMF

2) The tagmentation can be stopped by adding SDS to 0.1% and purified using a Zymo™ *DNA Clean and Concentration Kit* before agarose gel electrophoresis. It should be noted that the HS-Tn5 was highly resistant to guanidinium chloride (GuHCl) denaturation when compared to the original Tn5, which is common for protein fused to the elongation factor tsf. For the original Tn5, we could stop the tagmentation by adding equal volume of GuHCl containing Binding Buffer and use 1.8 X AMPure XP Beads to recover the DNA. We found this is not sufficient to strip HS-Tn5 from DNA. If AMPure XP Beads have to be used after HS-Tn5 tagmentation, we would add SDS to 0.1% (w/v), sufficient EDTA to chelate the Mg^{2+} and heat the sample 1 min at 65 °C to fully denature the HS-Tn5.

Preparation of transfection-grade plasmids

We found that the purification of the plasmid DNA and the size of the plasmid are two of the most critical factors for achieving high protoplast transfection efficiency. Hence, we generated a minimal cloning vector to express the TF, as well as a separated helper plasmid to express the Biotin ligase. After trying different commercial DNA preparation kits, we found that the Qiagen ones could generate endotoxin-free DNA for transformation. However, they are too costly, as each transformation requires hundreds of micrograms of DNA.

Therefore, we developed a silica dioxide (sand powder) method to isolate plasmid DNA followed by TritonX-114 based endotoxin removal, which yield 1-2 mg DNA at a relatively negligible cost.

Prepare TF constructions

1) Majority of our TF clones are obtained from maize TFome collection available through GRASSIUS in the form of Gateway Entry vectors[1]. These TF clones were transferred into our Gateway destination vector pHW-3xAvi (Addgene plasmid #127918) containing cauliflower mosaic virus 35S (CaMV35S) promoter and C-terminus 3xAvi tag by LR reaction. For 20 TFs that are not present in the clone collection, we PCR amplified their coding sequence from maize cDNA, and cloned them into the linearized pHW-3xAvi vector by Gibson assembly (Supplementary Table 1).

2) We have also PCR amplified the *E. coli* BirA gene and cloned it to a pUC19 plasmid with CaMV35S promoters. To avoid silencing of the transgenes, we also cloned the silencing suppressor p19 into the BirA plasmid to generate the plasmid pBirA-P19 (Addgene plasmid #127919), which were co-transformed into maize protoplast with the TF constructs.

Sand powder-based plasmid DNA maxiprep

1) To prepare the sand powder suspension (100 mg/mL), 5 g silicon dioxide (Sangon, A500823) was suspended thoroughly in 50 mL of ddH_2O and centrifuge at 1,000 g at room temperature for 15 min. The sand powder was washed two more times and resuspended in 50 mL of distilled water.

2) *E. coli* DH5α cells harboring the plasmid were grown overnight in 100 mL LB at 37°C overnight. Cells were spun down in 50 mL Falcon tubes, and the pellets were resuspended in 5 mL Buffer I by vortex.
   Buffer I:
   50 mM Glucose
   25 mM Tris-HCl pH 8.0
   10 mM EDTA

3) Next, 5 mL of Buffer II was added, and the tubes are mixed by gentle inversion.
   Buffer II:
   0.2 M NaOH
   1% SDS

4) After 2 min and the lysis reaction is neutralized by the addition of 5 mL Buffer III (3 M potassium acetate pH 4.8).

5) After centrifugation for 10 min at max speed, the supernatant was transferred to a clean Falcon tube. 2 μL RNase A (100 mg/mL stock) was added and incubated at 37°C for 15 min to digest the RNA. 5 mL of Chloroform was then added, and the solution was vortex and centrifuged at 4 °C at 4,000 x g for 5 min and the upper layer was transferred to a new Falcon tube with 3.5 g guanidine hydrochloride powder (2.5 M final). 500 μL of sand powder suspension from step (1) was added and mix well with rotation at room temperature for 30 min.

6) After 2 min centrifugation at 2,000 x g, the silica (sand) was resuspended in a new 2 mL centrifuge tube with 1 mL PB Buffer:
   5 M guanidine hydrochloride
   30% isopropanol

7) The silica was pelleted again and washed once using 1 mL PE Buffer:

16 mM NaCl
1.6 mM Tris-HCl pH 7.5
80% Ethanol

8) The silica was then washed once more with 1 mL of 80% Ethanol, centrifuged at maximum speed and dry in the 37°C incubator for 5 minutes.

9) The DNA was eluted twice with 1 mL of TE Buffer (10 mM Tris-HCl pH 8.0, 1 mM EDTA), and the silica was resuspended well by vortexed at a 37°C shaking incubator. The two elutes were then combined and the crude DNA concentration (~ 0.5 μg/μL) is checked by NanoDrop.

Removing endotoxin and concentrating the plasmid DNA

1) Transfer 1 mL of crude DNA from the previous step to a new 1.5 ml centrifuge tube with 0.2 mL (0.2 X volume) of Triton X and SDS Solution (6% (w/v) Triton X-114 and 3% (w/v) SDS)

2) The solution was then mixed by gentle inversion, incubated at room temperature for 10 min before 400 μL 4 M NaCl was added. The cloudy mixture was centrifuged at 12,000 g at room temperature for 10 min. The upper aqueous phase containing the endotoxin-free DNA was transferred to a new tube.

3) To precipitate the DNA, 2 μL of glycogen (20 mg/mL) and 1 volume of isopropanol were added, and the tube was incubated at -20°C for at least 1 hour before centrifugation at max speed for 20 min at 4°C.

4) The pellet was washed with 1mL of cold 80% ethanol, centrifuge for 10 min and air dried for 5-10 min. It was then dissolved in 200 μL ddH_2O and the DNA concentration is determined by NanoDrop. The concentration should be 1-5 μg/μL.

Transient expression of TF in leaf protoplasts

The traditional protoplast isolation methods often requires cutting leaves into 1-2 mm slices for cell wall digestion[2]. We were unable to improve the transformation efficiencies above 50% using protoplast isolated by these methods, and those protoplasts often have high fatality rate after overnight incubation. We realized that the excessive wounding to the plant tissues during the cell wall digestion is a cause of the low transformation efficiency. Hence, we developed a protoplast isolation method without the need of cutting the leaves. Instead, we peel the lower epidermis, which enables the cell wall digestion enzyme to gently penetrate the leaf and release the protoplasts. Video showing how to prepare the protoplasts has been uploaded online and could be viewed at https://www.bilibili.com/video/BV1M7411Z7m8/ and https://www.bilibili.com/video/BV1g7411Z7pL/.

1) The leaves on a 9-day-old seedling were first cut into large 2-3 cm sections, and their upper epidermis is attached to a masking tape. The midrib and the lower epidermis were then removed using tweezers. The extra masking tape is removal by scissors. Around 10 leaf disks with the exposed lower epidermis side down were then placed in the 90 mm petri dish with ~12 mL Enzyme Solution:
1% (w/v) CELLULASE ONOZUKA R-10
0.2% (w/v) MACEROZYME R-10
0.6 M Sorbitol
20 mM MES pH 5.5 (pH adjusted by KOH)
1 mM $MgCl_2$
1 mM $CaCl_2$
1 mM ß-mercaptoethanol

0.1% (w/v) BSA

Notes: We heat the enzyme solution without ß-ME and BSA at 55°C *for 10 min to inactivate DNase and proteases, which also help the enzymes to dissolve.*

2) The uncapped petri dish is then vacuumed for 15 seconds to allow the enzyme solution to penetrate the tissues. Digestion was performed on an orbital shaker at 50 rpm for not more than 4 hours at 25°C.

3) After digestion, the leaf disks were then flushed with a transfer pipettes to gently release the protoplasts from the tapes. Protoplasts were filtered through a 40 µm cell strainer and pelleted by centrifugation at 100 x g with "soft" setting for 3 min at 4 °C in a 14 mL round bottom falcon tube (Corning Cat#352059). The cell pellet was resuspended gently with 10 mL of cold Wash Buffer:
   2 mM MES pH 5.5 (pH adjusted by KOH)
   154 mM NaCl
   125 mM $CaCl_2$
   5 mM KCl

4) The cells were spun down, wash with chilled Wash Buffer again, and finally resuspended in 10 mL of chilled Wash Buffer. The tube was then left on ice for 30 min to allow the intact cells to sediment. The supernatant containing dead and broken cells were removed using a transfer pipette. The pellet was resuspended with 5 mL of chilled Transformation Buffer:
   4 mM MES pH 5.7 (pH adjusted by KOH)
   0.6 M mannitol
   15 mM $MgCl_2$

5) The cell number was counted on a hemocytometer, and more Transformation Buffer was added to bring the cell concentration to 10^6 cells/mL. The expected yield is ~ 10^7 cells from two 9-day-old seedlings at the 3-leaf-stage. Each transformation requires 1 mL of cells (~10^6). Once suspended in transformation buffer, the cells should be used immediately for transformation without delay. To save time, we often prepare the plasmid and PEG solution in advance.

6) In a new falcon tube, mix 100 µg of plasmid DNA (50 µg of TF-Avi plasmid and 50 µg of pBirA-P19). To check the transformation efficiency, we often add 5 µg of GFP plasmid as spike-in. 1 mL of protoplasts were gently added to the DNA and mixed by gentle pipetting with wide bore pipette tips. After 10 min incubation on ice, 1 mL of freshly prepared PEG Solution was added:
   40% (w/v) PEG 4000 (takes a few hours to dissolve on a rotator)
   0.2 M Mannitol
   0.1 M $CaCl_2$

7) The viscous solution was mixed by gentle rotation and tapping the tube. The cleared mixture was then incubated at room temperature for 15 min.

8) To stop the PEG transformation, 10 mL Wash Buffer was added and mixed by gentle inversion. The cells were pelleted by centrifugation for 3 min at 100 x g with "soft" setting at room temperature, washed one more time with 10 mL Wash Buffer.

9) The cells were finally resuspended in 20 mL Wash Buffer with 100 ug/mL Timentin (100 mg/mL) and 1 µM biotin (50 mM in DMSO) and gently poured onto a 90 mm petri dish precoated with 0.1% BSA for overnight incubation in the dark at RT.

TF biotin ChIP-Seq

Cross-linking, nuclear lysis and DNA shearing

1) After overnight incubation, protoplasts were fixed for 5 minutes in the petri dish with the adding of 1/4 volume of Fixation Buffer:
   4% (w/v) Formaldehyde
   154 mM NaCl
   125 mM $CaCl_2$

2) To quench the formaldehyde, add 1/10 volume of Stop Buffer:
   2 M glycine
   100 mM Tris-HCl pH 8.0

3) Protoplasts were then transferred to a centrifugation tube and spun down at 3,000 g for 2 min at 4°C and resuspended in 1 mL cold Lysis Buffer:
   10 mM Tris-HCl pH 8.0
   1 mM EDTA
   0.1% (w/v) SDS
   1 mM ß-ME
   1 X protease inhibitor

4) The released nuclei were transferred to an eppendorf tube, spun down and washed multiple times with cold Lysis Buffer till the nuclei pellet were no longer dark green, which indicates the chloroplasts have been completely lysed.

5) The washed nuclei were resuspended in 100 μL Sonication Buffer and transferred to a Covaris microtube or 300 μL for sonication in Bioruptor.
   10 mM Tris-HCl pH 8.0
   1 mM EDTA
   0.2% (w/v) SDS
   1 X protease inhibitor
   0.1 mg/mL BSA

6) The chromatin was fragmented using the Covaris M220 (10% duty factor, 5 min) or Bioruptor (15 cycles, high power, 30/30 ON/OFF), and the insoluble debris was removed by centrifugation at maximum speed.

7) The insoluble debris was removed by centrifugation at maximum speed for 5 min. The supernatant containing the sonicated chromatin was transferred to a 1.5 mL centrifuge tube. The SDS was quench by added equal volumes of 2% Triton X-100.

Biotin Pull-Down and tagmentation for Illumina sequencing

1) Transfer 15 μL MyOne™ Streptavidin T1 Dynabeads™ to a PCR tube and recover the beads on magnet. Wash the beads once by adding 100 μL HS (High Salt) Buffer:
   1 M NaCl
   10 mM Tris-HCl pH 8.0
   1 mM EDTA
   1% Triton X-100
   1 X PMSF

2) Wash twice with 100 μL TE-Urea Buffer:

    2 M Urea
    10 mM Tris-HCl pH 8.0
    1 mM EDTA
    0.1% Tween-20
    1 X PMSF

3) After washing the beads twice with 100 μL HS buffer, resuspend the beads using 0.5 mL HS Buffer and add to the sheared chromatin. Incubate on a rotator at 4°C for >3 hours.

4) Wash the bound beads five times with 0.5 mL HS Buffer and transferred to a new tube.

5) Wash the bound beads twice with 0.5 mL TE-Urea buffer and transferred to a new tube.

6) Wash the bound beads twice with 0.1 mL TET (TE pH8.0 with 0.1% Tween-20) and transfer to a PCR tube. Finally, beads were resuspended in 40 μL of Tagmentation Buffer:

    10 mM Tris-HCl pH 8.0
    10 mM $MgCl_2$
    15% DMF

7) To each tube, 0.5 μL 11 uM (~1 OD_{280}) assembled HS-Tn5 was added and mixed by pipetting. The reaction was incubated for 30 min on a rotator in the 37°C oven.

8) After tagmentation, the beads were washed twice with HS buffer, TE-Urea Buffer and SDS Buffer (TE pH 8.0 with 0.2% (w/v) SDS), and finally resuspended in 50 μL Reverse Crosslink Buffer:

    0.3 M NaCl
    10 mM TE pH 8.0
    0.1% (w/v) SDS
    1 μL lambda DNA (10 ng/μL)
    1 μL proteinase K

9) The tubes were incubated in a PCR machine at 55°C for 1 hour and 63°C for 4 hours. After reverse-crosslinking overnight, the tube was placed on the magnet and the supernatant is transferred to 500 uL Binding Buffer in a spin column (Zymo™ *DNA Clean and Concentration Kit*). The beads were washed again with 50 μL TE and the supernatant was added to the Binding Buffer. The purified DNA was PCR amplified with oligo matching the Illumina Nextera primers with i5 and i7 indexes. Around 50 libraries were pooled for low-depth sequencing in one HiSeq X lane, and the libraries that passed ENCODE2 QC pipeline were chosen for further sequencing according to their unique read ratio.

Supplementary Method 2. Bundle sheath and mesophyll RNA-seq protocol

Bundle sheath strands can be isolated from remaining leaf tissue after the enzyme digestion following the steps in 1.3. After the enzyme digestion, the protoplasts solution was filtered by a 40 um cell strainer, and the bundle sheath strands were retained on the cell strainer surface. The cell strainer was then transfer to a new petri dish and washed with 10 mL cold Wash Buffer with 0.1% Triton X-100 multiple times. The dish was gently shaken at 100 rpm to wash off the remaining mesophyll cells. The bundle sheath strands were visually inspected under the microscope to confirm the complete removal of mesophyll cells. The strands can be used directly or frozen in liquid nitrogen. The mesophyll cell pellet and the washed bundle sheath strands are grounded in pre-cooled 1.5 mL Eppendorf tube containing 450 µL RLT Buffer with ß-mercaptoethanol (RNeasy Plant Mini Kit, QIAGEN), and the subsequent RNA purification procedures were performed following the instruction outlined by manufacturer. To prepare the RNA-Seq library for Illumina Sequencing, we follow the non-strand-specific TruSeq RNA Library Prep protocol, during which the second strand cDNA was synthesized using dNTP as previous described[3].

Supplementary Method 3. Histone ChIP-seq protocol

Tissue fixation and lysis

1) 10^6 mesophyll cells was fixed and lysed following the steps in Note 1.3. For the bundle sheath strands, the washed strands were vacuum in fixation buffer at room temperature for 10 min:
   1% (w/v) Formaldehyde
   0.6 M sorbitol
   10 mM $MgCl_2$
   10 mM KCl

2) The fixation reaction was stopped by adding the Glycine Solution as previously described, followed by 5 min vacuum at room temperature. After quenching the formaldehyde, the buffer was drained, and the strands were recovered using cell strainer.

3) The bundle sheath strands were washed once and resuspended in 5 mL chilled Wash Buffer. SDS was added to 0.2% and the strands were incubated on a rotator at 4°C for 10 to 20 minutes to lyse the chloroplasts and release the nuclei from the strand.

4) The empty strands were then removed by passing a fresh 40 μm cell strainer and the nuclei were collected from flow-through by centrifugation under 2,000 g at 4°C for 5 min. Nuclei pellets of mesophyll cells and bundle sheath cells were washed 2-3 times with Lysis Buffer.

Chromatin shearing and immunoprecipitation

1) The washed nuclei were resuspended in the 120 μL Sonication Buffer and transferred to a Covaris microTube. The chromatin was fragmented using the Covaris M220 (5% duty factor, 5 min), and the insoluble debris was removed by centrifugation at full speed. The chromatin was diluted to 500 μL with Low Salt Buffer:
   150 mM NaCl
   1% Triton X-100
   1 mM EDTA
   10 mM Tris-HCl pH 8.0
   1 X protease inhibitor

2) To pre-clear the chromatin, washed protein A or G Dynabeads in 100 μL Low Salt Buffer was added to the chromatin and the tubes were incubated on rotator at 4°C for at least one hour. The pre-cleared chromatin was recovered from the magnetic stand and spin at maximum speed for 5 min to further remove the residue beads and insoluble debris.

3) The histone modification antibodies (RevMAb and Millipore) were incubated with suitable protein A/G Dynabeads in Low Salt Buffer for at least 3 hours and then added to the pre-cleared chromatin sample for immunoprecipitation overnight.

4) The beads were washed twice with Low Salt Buffer and transferred to a new tube.

5) The beads were then washed twice with High Salt Buffer and transferred to a new tube.
   500 mM NaCl
   1% Triton X-100
   1 mM EDTA
   10 mM Tris-HCl pH 8.0

6) Wash the beads two more times using LiCl Buffer:
   250 mM LiCl
   1% Triton X-100
   1 mM EDTA
   10 mM Tris-HCl pH 8.0

7) Finally wash twice using Tris Buffer:
   20 mM Tris-HCl pH 8.0
   0.2% Tween

After washing, the beads were resuspended in 40 µL of Tagmentation Buffer with of 0.5 µL 11 uM (~1 OD_{280}) assembled Tn5, and incubated on a rotator at 37°C for 30 min. After Tn5 tagmentation, the Dynabeads were washed twice with Low Salt Buffer, High Salt Buffer and TE. Finally, the beads were resuspended in 100 µL Reverse Crosslink Buffer. The tubes were incubated in a PCR machine at 55°C for 1 hour and 63°C for 4 hours. After reverse-crosslinking, the supernatant is recovered on a magnetic rack, mixed with 500 µL Zymo™ Binding Buffer, and the DNA was purified using Zymo™ *DNA Clean and Concentration Kit*. The library was then PCR amplified with oligo matching the Illumina Nextera primers with i5 and i7 indexes.

Supplementary Method 4. ATAC-seq protocol

1) Wash the leaf using ddH_2O with 0.01% TritonX-100. Wipe off excessive water with tissue, and fold the leaves prior to cutting in 10 mL Sorbitol Cutting Buffer:

    0.6 M sorbitol
    20 mM MES pH 5.7 (pH adjusted by KOH)
    20 mM KCl
    10 mM $MgCl_2$
    1 X PMSF
    0.2% Triton X-100
    1 mM ß-mercaptoethanol
    0.1 mg/mL BSA
    1 X proteinase inhibitor

2) To collect the nuclei, we first filtered the nuclei solution using the 40 μm cell strainer, used a dropper transfer the flow-through to 13 mL round bottom tube and spin down 10 min with 500 g at 4°C. The nuclei pellet was then resuspended with 1 mL Sorbitol Cutting Buffer and filtered again using a 10 μm Nylon mesh. The nuclei were spun down, washed once with 150 uL Sorbitol Cutting buffer and transferred to a new PCR tube. Finally, the nuclei was washed once with 150 uL BR Tn5 buffer:

    20 mM Tris-HCl pH 7.8
    10 mM $MgCl_2$
    60 mM KAc

3) The nuclei was spun down at 500 g at 4°C for 2 min, and resuspend with 50 μL BR Tagmentation Buffer with DMF:

    20 mM Tris-HCl pH 7.8
    10 mM $MgCl_2$
    60 mM KAc
    15% DMF

4) For ~5 x10^5 nuclei, we added 1 μL 11 uM (1 OD_{280}) Tn5 and incubate the PCR tube at 37°C for 30 min on a rotator. To stop the transposition, add 100 μL Stop Buffer:

    1 X PBS
    25 mM EDTA
    1% Triton X-100

5) The nuclei were spun down at 2,000 g at room temperature for 1 min. The pellete was resuspended the in 20 μL Lysis Buffer (TE pH 8.0 with 0.1% SDS).

6) DNA was purified using Zymo™ *Select-a-Size DNA Clean & Concentrator Kits*. The DNA could be PCR amplified with oligo matching the Illumina Nextera primers with i5 and i7 indexes.

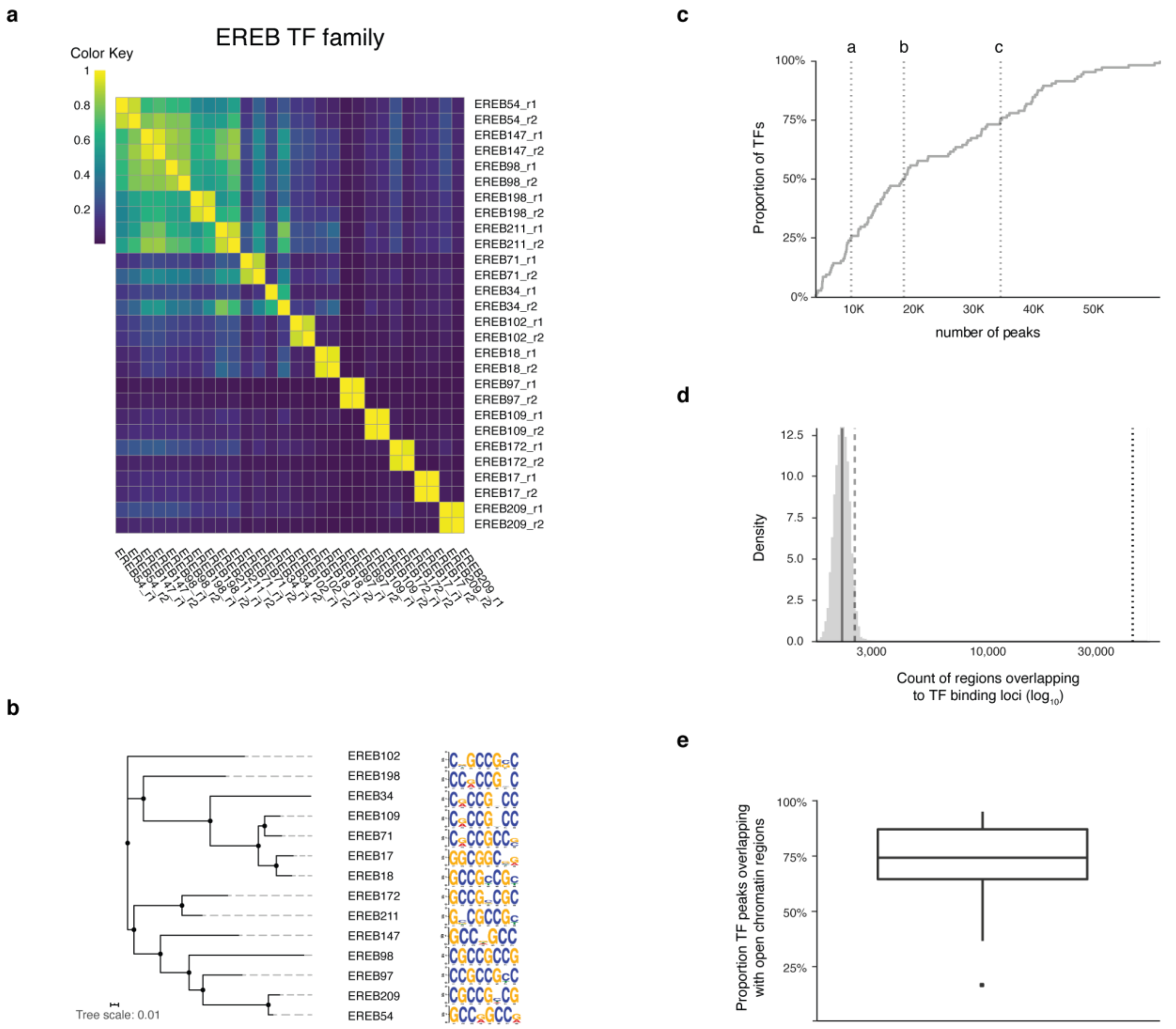

Supplementary Figure 1. Correlation between replicates and TF binding overlap with open chromatin regions. a, Pearson correlation of biological replicates of 14 EREB TFs. **b**, Motif of the 14 EREB TFs. **c**, Cumulative distribution function of number of total peaks observed for each of the 104 TFs. The 25th, 50th, and 75th percentiles are indicated with dotted lines, and labeled a-c respectively. **d**, TF binding loci significantly overlap with open chromatin regions. Density plot showing the overlap of TF binding loci positions and open chromatin regions (dotted line), versus an empirical null distribution from circular randomized (n=10,000 permutations) positions of TF binding loci. Mean and alpha value of 0.05 for the empirical null indicated with a solid grey line and dashed line respectively. **e**, Box plot shows the distribution of the percentage of TF (n=104) total peaks that overlap with open chromatin regions. The interquartile region (25th-75th) correspond to the range between 64%-87% with minima 16.67%, maxima 95.09%, center is the median value 74.25%.

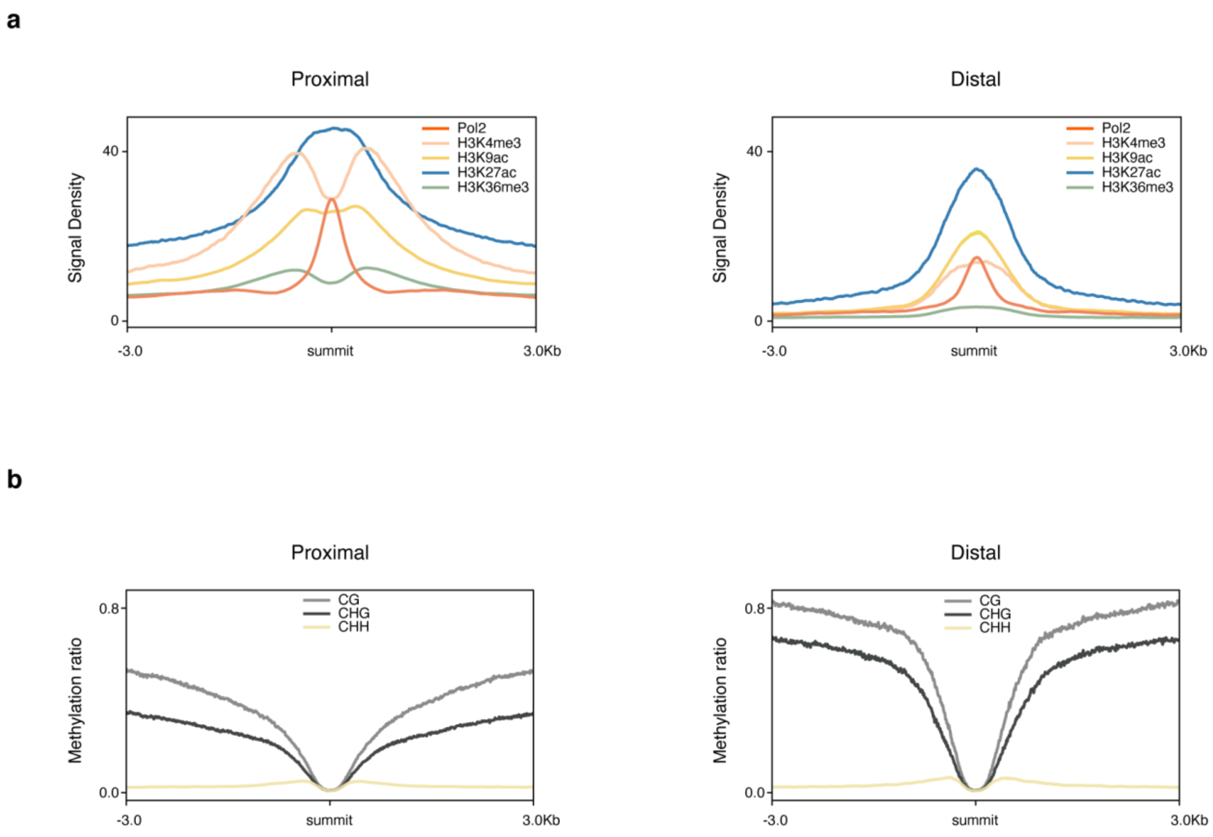

Supplementary Figure 2. Epigenome status of the proximal and distal TF binding sites. We measure DNA methylation in three different sequence contexts, as well as different chromatin marks such as H3K4me3, H3K9ac, H3K27ac, H3K36me3 and RNA Polymerase II ((Pol2). ZmbHLH43 is one of the TF that binds to a known distal regulatory region *Vgt1*. We then isolate its proximal binding sites (2kb upstream of genes) and distal binding sites (5kb away from genes). The epigenome feature signal density plots showed similar enrichment patterns for both proximal and distal sites.

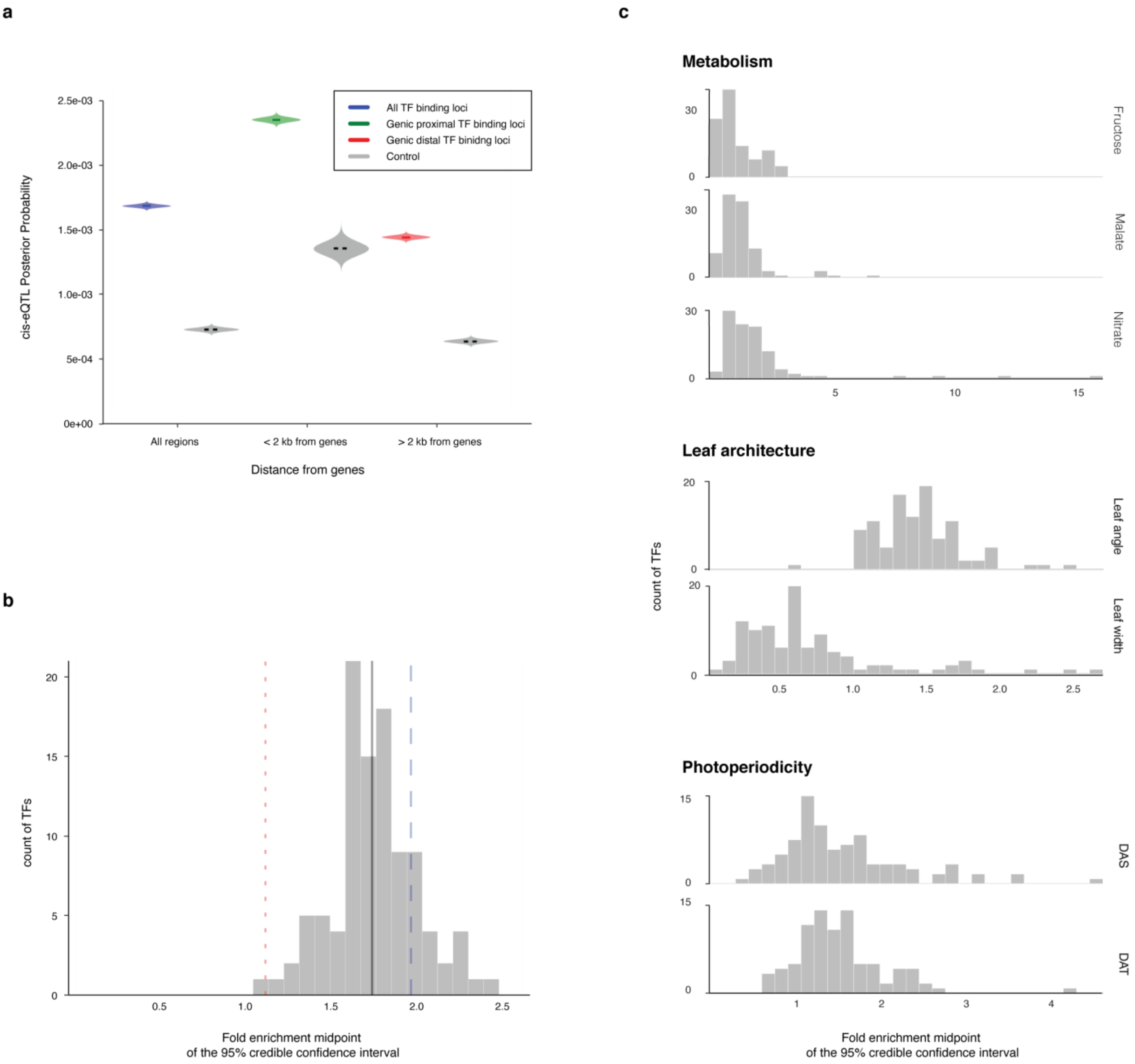

Supplementary Figure 3. TF binding associated with eQTL and GWAS hits. a, Posterior probability (on the y axis) that the highest significance SNP of an eQTL overlaps a TF peaks (n=2,147,346) versus flanking regions. Results for (a) all the peaks, (b) only peaks nearby genic regions (genic regions as annotated in the reference genome v4) and (c) only peaks distal from genic regions were plotted. shows posterior probability. The center values correspond to the medians of the distributions. **b**, A large number of individual TFs show enrichment in cis-eQTLs. Distribution of the midpoint of the 95% confidence interval for the relative enrichment of leading cis-eQTL SNPs for each of the TFs. Vertical bars show the values for introns (red, dotted line), 5’UTRs (blue, dashed line), and average of the TFs (grey, solid line). **c**, TFs binding show enrichment in GWAS hits for complex multi-gene traits. Distribution of the midpoint of the 95% confidence interval for the relative enrichment of GWAS-hits for each of the TFs in seven traits (Metabolites: Fructose, Malate, Nitrate; Leaf architecture: Leaf angle, Leaf width; Photoperiodicity: Days to silking, Days to anthesis).

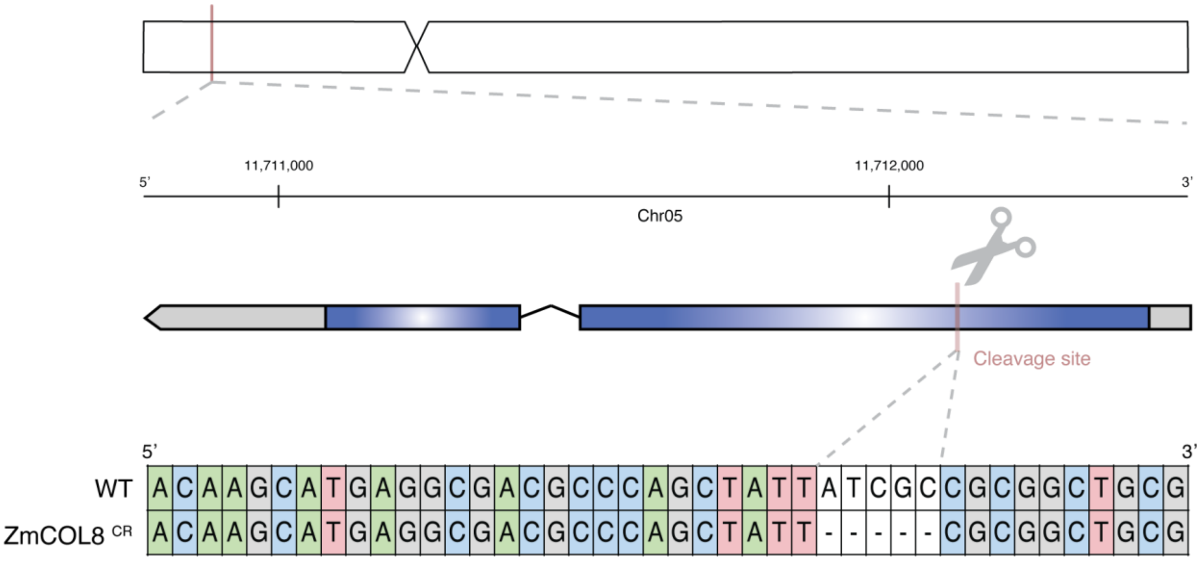

Supplementary Figure 4. The COL8 CRISPR-Cas9 mutation. Without a natural COL mutant in maize, we searched different maize CRISPR/Cas9 populations, and found one COL8 knockout line from the maize transcription factor CRISPR/Cas9 knockout pool generated by Wimi Biotech Co. Ltd. We screened the seeds from Wimi Biotech and found this line carries a frame-shift deletion in the COL8 gene coding region in the first exon. The homozygous mutant has a pale-green phenotype and seedling lethality, and the line have to be maintained in the heterozygous form.

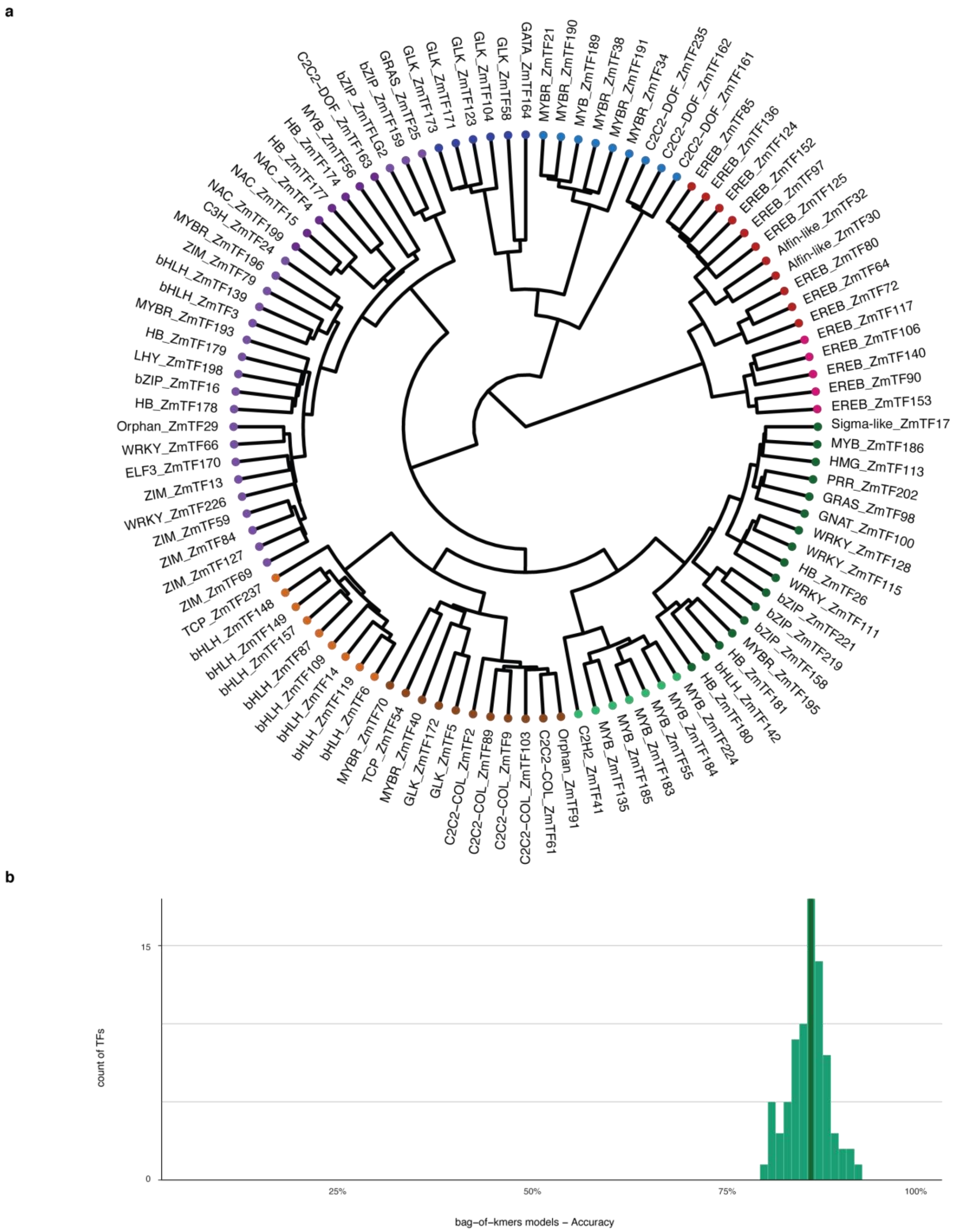

Supplementary Figure 5. Machine learning bag-of-k-mer models. a, Sequence preferences learned by the model can recapitulate TF family relationship. Dendrogram shows 104 TF clustered by sequence binding similarity (predicted k-mer weights) derived from the sequence models, and colors indicate the cluster in the tree. **b**, Sequence models can predict TF binding preferences. Histogram of the average accuracy values (5 folds cross-validation) obtained with the bag-of-k-mer models in the prediction of individual TF binding. Mean value is indicated with a green solid line.

a

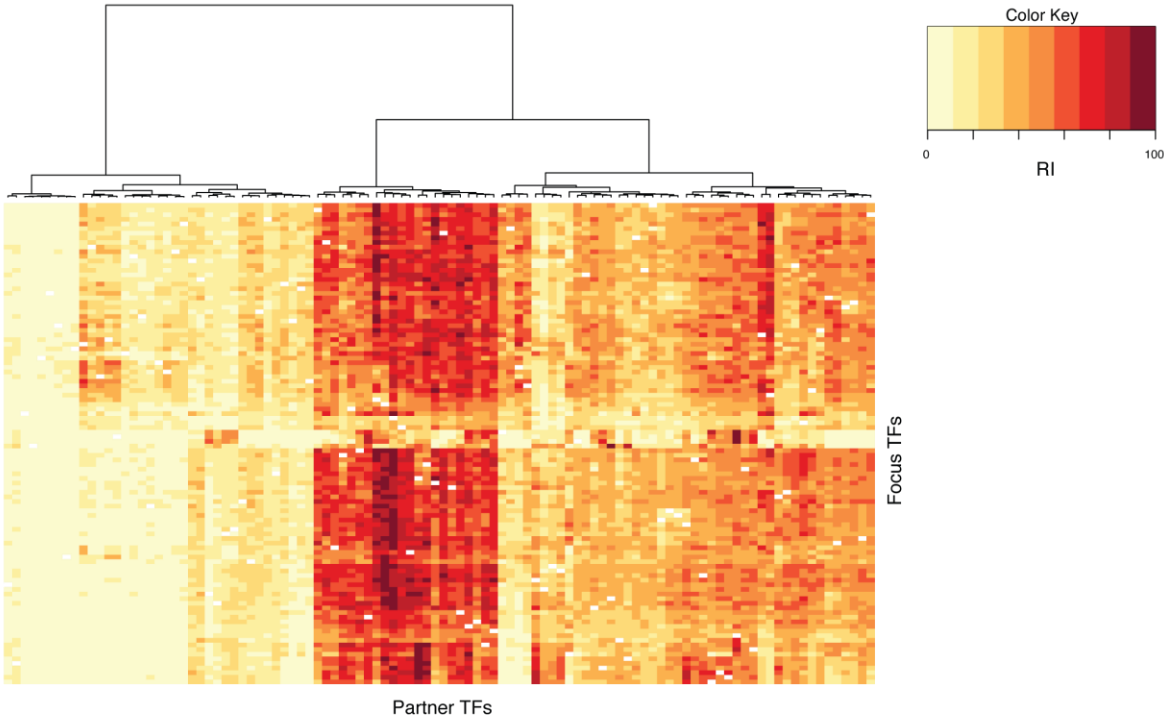

b

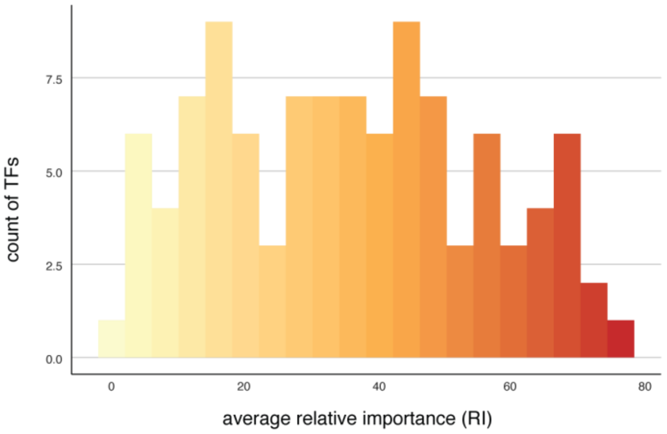

Supplementary Figure 6. TF co-localization models predict TF binding preferences. a, Heatmap summarizing the co-localization model. The visualized matrix is composed of stacked average relative importance scores (RI values), for each of TF as a focus TF (rows) and TF as partners (columns). Rows and columns are clustered to highlight regulatory patterns predicted from the co-localization models. **b**, Distribution of the "combinatorial potential" for each TF (average RI values across context).

a

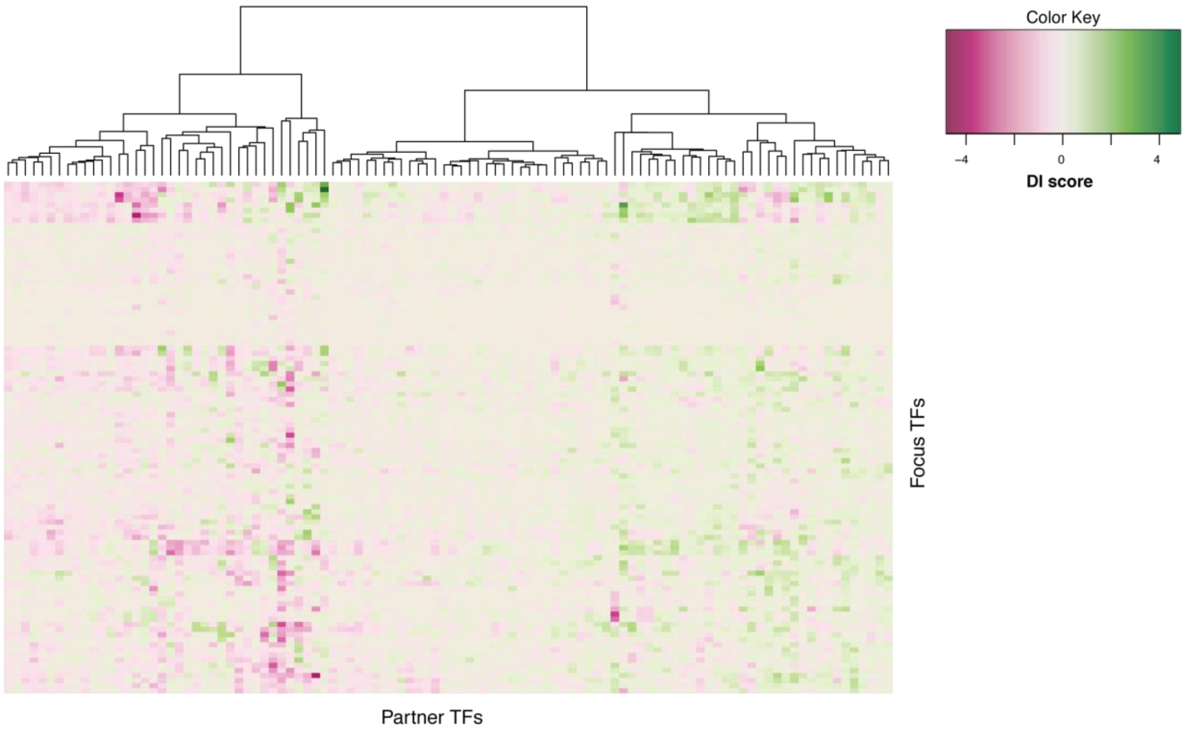

b

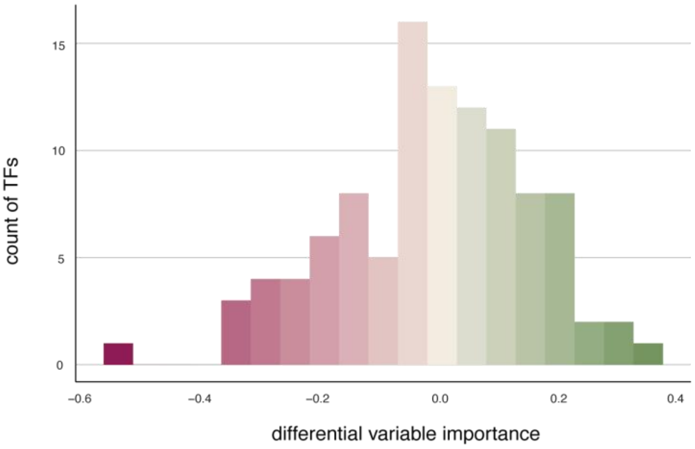

Supplementary Figure 7. Co-localization model predicts differences in co-localization patterns between genic proximal and distal regions. a, Heatmap summarizing the differential importance score between genic proximal, and distal regions. The visualized matrix is composed of stacked average differential importance scores (DI values), for each of TF as a focus TF (rows) and TF as partners (columns). Rows and columns are clustered to highlight patterns of regulation in the genic or distal regions. **b**, Distribution of the "localization bias" for each TF (average DI values across context).

Supplementary References

1. Burdo, B. *et al.* The Maize TFome – development of a transcription factor open reading frame collection for functional genomics. *Plant J* **80**, 356–366 (2014).
2. Sheen, J. Signal transduction in maize and Arabidopsis mesophyll protoplasts. *Plant Physiology* **127**, 1466–1475 (2001).
3. Zhong, S. *et al.* High-throughput Illumina strand-specific RNA sequencing library preparation. *Cold Spring Harb Protoc* **2011**, pdb.prot5652 (2011).
