# Supplementary material for: Reconstructing the maize leaf regulatory network using ChIP-seq data of 104 transcription factors
Source: Nat Commun. 2020 Oct 9;11:5089. doi: 10.1038/s41467-020-18832-8 (PMC7547689; doi:10.1038/s41467-020-18832-8)
Supplement: Supplementary file 3 — Reporting Summary [file 41467_2020_18832_MOESM3_ESM.pdf]

nature research

Corresponding author(s): Silin Zhong, María Katherine Mejía-Guerra, Pinghua Li

Last updated by author(s): 09/09/2020

Reporting Summary

Nature Research wishes to improve the reproducibility of the work that we publish. This form provides structure for consistency and transparency in reporting. For further information on Nature Research policies, see our Editorial Policies and the Editorial Policy Checklist.

Statistics

For all statistical analyses, confirm that the following items are present in the figure legend, table legend, main text, or Methods section.

n/a	Confirmed	
☐	☒	The exact sample size (*n*) for each experimental group/condition, given as a discrete number and unit of measurement
☐	☒	A statement on whether measurements were taken from distinct samples or whether the same sample was measured repeatedly
☐	☒	The statistical test(s) used AND whether they are one- or two-sided<br>*Only common tests should be described solely by name; describe more complex techniques in the Methods section.*
☒	☐	A description of all covariates tested
☐	☒	A description of any assumptions or corrections, such as tests of normality and adjustment for multiple comparisons
☐	☒	A full description of the statistical parameters including central tendency (e.g. means) or other basic estimates (e.g. regression coefficient) AND variation (e.g. standard deviation) or associated estimates of uncertainty (e.g. confidence intervals)
☐	☒	For null hypothesis testing, the test statistic (e.g. F, t, r) with confidence intervals, effect sizes, degrees of freedom and P value noted<br>*Give P values as exact values whenever suitable.*
☒	☐	For Bayesian analysis, information on the choice of priors and Markov chain Monte Carlo settings
☒	☐	For hierarchical and complex designs, identification of the appropriate level for tests and full reporting of outcomes
☒	☐	Estimates of effect sizes (e.g. Cohen's d, Pearson's r), indicating how they were calculated

Our web collection on statistics for biologists contains articles on many of the points above.

Software and code

Policy information about availability of computer code

Data collection	TF ChIP-seq libraries were prepared and sequenced on Illumina HiSeq X10 Sequencing System with manufacture's instruction.
Data analysis	Open source softwares used include: Bowtie2 (version 2.2.5), SAMTools (version 1.3), deepTools (version 3.2.0), PhantomPeakQualTools (version 1.14), BEDTools (version 2.27.1), IDR (Irreproducible discovery rate) statistical framework (version 2.0.3), MACS2 (version 2.1.2), HapMap (version 3.2.1), VCFtools (version 0.1.17), PLAZA (version 4.0 Monocots), MEME suite (version 5.0.5), RuleFit3 algorithm as well as R version 3.6.1 (2019-07-05) and Python to analyze all the datasets. Details about the usages and the parameters of the softwares were stated in the manuscript. The command and R scripts used have been uploaded to https://bitbucket.org/bucklerlab/p_transcriptionfactors/src/master/Analyses/ . The bag-of-k-mers models have been uploaded to Zenodo as a dataset with a doi https://zenodo.org/record/3834199.

For manuscripts utilizing custom algorithms or software that are central to the research but not yet described in published literature, software must be made available to editors and reviewers. We strongly encourage code deposition in a community repository (e.g. GitHub). See the Nature Research guidelines for submitting code & software for further information.

Data

Policy information about availability of data

All manuscripts must include a data availability statement. This statement should provide the following information, where applicable:

- Accession codes, unique identifiers, or web links for publicly available datasets
- A list of figures that have associated raw data
- A description of any restrictions on data availability

Sequencing data have been deposited in the NCBI SRA database under the accession number PRJNA518749. Processed data have been deposited in the NCBI GEO database under the accession number GSE137972, and will be incorporated into the future MaizeGDB release.

Field-specific reporting

Please select the one below that is the best fit for your research. If you are not sure, read the appropriate sections before making your selection.

[x] Life sciences [ ] Behavioural & social sciences [ ] Ecological, evolutionary & environmental sciences

For a reference copy of the document with all sections, see nature.com/documents/nr-reporting-summary-flat.pdf

Life sciences study design

All studies must disclose on these points even when the disclosure is negative.

Sample size	No statistics method is used to determine sample size for TF ChIP-seq. Because it is not a population study and the important issue is that the biological samples are accurately stages for comparison, not the sample size. We have preformed ChIP-seq for 104 TFs, and each has no less than 2 biological replicates for peak calling. The information for each experiments is listed in Sup. Table 1.<br><br>The eQTLs were obtained from a published study (Kremling et al., Nature, 2018 – DOI: 10.1038/nature25966). In brief, the sample size (mean 255 individuals per tissue) to collect RNA-seq was considered enough as previous studies (i.e., the GTEx project) have shown the ability to map eQTLs with < 300 individuals. The phenotype data used in this study for the GWAS analysis was all derived from previous studies on the US-NAM population. In brief, the GWAS dataset consisted on traits measured on 5,000 mapping lines derived from 25 related crosses (the US-NAM population). The US-NAM population has been studied for the past 20 years, and proven a powerful tool to map complex traits. McMullen et al., Science, 2009 - DOI: 10.1126/science.1174320
Data exclusions	No data was excluded from the analysis
Replication	All replication were reliably reproduced in multiple independent experiments as indicated in the Supplementary Table 3.
Randomization	Randomization was not used.
Blinding	No blinding was used.

Reporting for specific materials, systems and methods

We require information from authors about some types of materials, experimental systems and methods used in many studies. Here, indicate whether each material, system or method listed is relevant to your study. If you are not sure if a list item applies to your research, read the appropriate section before selecting a response.

Materials & experimental systems

n/a	Involved in the study	
[ ]	[x]	Antibodies
[x]	[ ]	Eukaryotic cell lines
[x]	[ ]	Palaeontology and archaeology
[x]	[ ]	Animals and other organisms
[x]	[ ]	Human research participants
[x]	[ ]	Clinical data
[x]	[ ]	Dual use research of concern

Methods

n/a	Involved in the study	
[ ]	[x]	ChIP-seq
[x]	[ ]	Flow cytometry
[x]	[ ]	MRI-based neuroimaging

Antibodies

Antibodies used	Antibodies used for Histone ChIP-seq: Anti-trimethyl H3K27 (1 µg/µl, Millipore, 07-449), anti-trimethyl H3K4 (1 µg/µl, Millipore, 05-745R)
Validation	The antibodies have been validated by the company using dot plot, western plot, immuno-fluorescence, ChIP-qPCR experiments. Further validation report could be found on the the supplier website.

ChIP-seq

Data deposition

[x] Confirm that both raw and final processed data have been deposited in a public database such as GEO.

[x] Confirm that you have deposited or provided access to graph files (e.g. BED files) for the called peaks.

Data access links<br>*May remain private before publication.*	Sequencing data have been deposited in the NCBI SRA database under the accession number PRJNA518749. Processed data have been deposited in the NCBI GEO database under the accession number GSE137972.
Files in database submission	The information of 238 sequencing datasets involving in the study are listed in Supplementary Table 3.<br>15 data files:<br>Supplementary Data 1: 104 TFs selected for biotin ChIP-seq<br>Supplementary Data 2: Quality control information of the ChIP-seq libraries<br>Supplementary Data 3: List of all ChIP-seq and ATAC-seq libraries<br>Supplementary Data 4: Pearson correlation of ChIP-seq library replicate bam files<br>Supplementary Data 5: TF binding loci in maize leaf<br>Supplementary Data 6: TF GO-term and MAPMAN functional category enrichment<br>Supplementary Data 7: The hub genes and the high TF occupancy regions<br>Supplementary Data 8: eQTL enrichment<br>Supplementary Data 9: GWAS enrichment<br>Supplementary Data 10: Gene regulatory network<br>Supplementary Data 11: Network modules<br>Supplementary Data 12: Performance of bag-of-k-mers models<br>Supplementary Data 13: Results of bag-of-k-mers models<br>Supplementary Data 14: Results of co-localization models<br>Supplementary Data 15: Performance of co-localization models
Genome browser session<br>(e.g. UCSC)	http://137.189.43.55/JBrowse/?data=maize

Methodology

Replicates	Each sample with at least two biological replicates with high overlap for ChIP-seq peaks between replicates in each sample. All replicates generated for each of the 104 TF ChIP-seq experiments were listed in Supplementary Tables 3.
Sequencing depth	20 million pair-end raw reads for each sample with the length of 150bp. The detailed information as well as mapping statistics and peak calling results for each ChIP-seq datasets were listed in Supplementary Table 1.
Antibodies	Antibodies used for Histone ChIP-seq: Anti-trimethyl H3K27 (1 μg/μl, Millipore, 07-449), anti-trimethyl H3K4 (1 μg/μl, Millipore, 05-745R)
Peak calling parameters	SPP peak caller was used with relaxed parameters: "-i = <INPUT BAM FILE>", "-npeak = 300000". Before determining reproducible peak calls, we subtracted a list of genome blacklist regions, together with peaks located to mitochondria/chloroplast produced from SPP using the BEDTools. Reproducible peak calls were obtained with the IDR statistical framework, as implemented in the PhantomPeakQualTools package, using 1% IDR as a threshold (cut-off). Detail information regarding the data quality control, commands for peak calling, and the parameters used were described in the Methods.
Data quality	We followed ENCODE2 pipeline to evaluate our data. Biological replicates with Pearson correlation coefficient > 0.8 were retained for further analyses. To assess the signal-to-noise ratios based on normalized strand cross-correlation coefficient (NSC >= 1.05) and relative strand cross-correlation coefficient (RSC >= 0.8) on the individual alignment files from high reproducible libraries, PhantomPeakQualTools was used. We also visualized peak signals on genome browser for each dataset.
Software	Bowtie2, SAMTools, deepTools, PhantomPeakQualTools, BEDTools, IDR (Irreproducible discovery rate) statistical framework, MACS2, HapMap , VCFtools, PLAZA, MEME suite, RuleFit3 algorithm.
