# Supplementary material for: Reconstructing the maize leaf regulatory network using ChIP-seq data of 104 transcription factors
Source: Nat Commun. 2020 Oct 9;11:5089. doi: 10.1038/s41467-020-18832-8 (PMC7547689; doi:10.1038/s41467-020-18832-8)
Supplement: Supplementary file 4 — Description of Additional Supplementary Files [file 41467_2020_18832_MOESM4_ESM.pdf]

**Description of Additional Supplementary Files**

File name: Supplementary Data 1:
Description: The 104 maize leaf TFs selected for ChIP-seq

File name: Supplementary Data 2:
Description: Quality control information of the ChIP-seq libraries

File name: Supplementary Data 3:
Description: List of ChIP-seq and ATAC-seq libraries

File name: Supplementary Data 4:
Description: Pearson correlation of ChIP-seq library replicate bam files

File name: Supplementary Data 5:
Description: TF binding loci in the maize leaf

File name: Supplementary Data 6:
Description: TF classification based on their target gene GO-term and MAPMAN functional category enrichment

File name: Supplementary Data 7:
Description: The hub genes and the high TF occupancy regions

File name: Supplementary Data 8:
Description: eQTL enrichment in TF binding sites

File name: Supplementary Data 9:
Description: GWAS hit enrichment in TF binding sites

File name: Supplementary Data 10:
Description: The gene regulatory network

File name: Supplementary Data 11:
Description: Network modules

File name: Supplementary Data 12:
Description: Performance of bag-of-k-mers models

File name: Supplementary Data 13:
Description: Results of bag-of-k-mers models

File name: Supplementary Data 14:
Description: Performance of co-localization models
