# Supplementary material for: Reconstructing the maize leaf regulatory network using ChIP-seq data of 104 transcription factors
Source: Nat Commun. 2020 Oct 9;11:5089. doi: 10.1038/s41467-020-18832-8 (PMC7547689; doi:10.1038/s41467-020-18832-8)
Supplement: Supplementary file 2 — Peer Review File [file 41467_2020_18832_MOESM2_ESM.pdf]

Reviewer #1 (Remarks to the Author):

Overall, this is a very interesting set of experiments that have been clearly described and fairly interpreted. The collection of ChIPseq experiments provide a substantial new body of empirical data that will be very useful to the plant research community. The additional analyses support the validity of the data, and provide additional support for combinatorial models of regulation of transcription.

I have only two scientific comments. First, aside from the eQTL analysis and perhaps some coexpression analysis, its not clear how extensively expression data has been used to interpret these ChIPseq results on a genome wide scale, which would have the potential to provide some additional biological insight. Relatedly and more specifically, the use of GRNs with conditional expression analysis may be informative. It would also potentially help to improve GRNs with other TFs not included in this dataset to compare their empirical results with GRNs that were constructed naive of the ChIP datasets for these 104 TFs.

Also, I note the following grammatical/typographical issues:
P1 line 26 missing word:
complex agronomic traits. Machine-learning analyses were used to TF sequence preferences, and
line 42, eukaryotes?
unicellular model for eukaryote,

line 57 unnecessary comma
elements in the accessible chromatin ,and the TFs that bind to them

line 58 grammar…despite the fact that? Use "Although TF binding information…."?
Despite TF binding information is crucial for understanding how genes are regulated,

Line 60 unnecessary article "…express epitope fusion proteins" rather than "…the epitope fusion protein"

Line 63 should be "that are expressed" because talking about 104 TFs (plural)

Line 69 Use of instead of use

Line 71 perform ChIP-seq not performed

Typo on Manhattan Plot (Fig 2d) GAWS instead of GWAS

Line 175 I believe network should be plural in each usage (networks).

Reviewer #2 (Remarks to the Author):

The authors developed a high-throughput ChIP-seq protocol for Maize leaf protoplast and applied it to produce 218 ChIP-seq experiments for 104 TFs expressed in Maize leaf. They show that TF peaks are enriched in open chromatin, GWAS, and eQTL regions, and that there is a reduction in sequence variants in Maize strains at TF peak centers. Their primary use of the data set is for network analysis. First, they describe global properties of network modules, and then go into greater detail exploring TF modules implicated by their analysis to be involved in photosynthesis including mutant analysis of on factor. Finally, using ML they predict TF co-binding partners.
Overall it is a good study consisting of a new high-throughput approach for plant ChIP-seq, a major

laboratory feat to perform 218 TFs, and a valuable resource for the community. The analysis is good but could be improved. The primary issues I had is that they do not provide sufficient QC analysis of the ChIP, some figures can be improved for greater clarity and additional explanation of results is warranted in some cases, and more space could be provided for analysis of TF family/individual TF properties. Of these, better QC analysis of the ChIP-seq is most critical as it is difficult as it is presented to judge the data quality making it harder to fully assess the quality of the analysis and the resource.

Major issues:

1. The authors provide a very detailed protocol in the supplementary material, but the manuscript would benefit from a little more detail in the main text and figure to accompany it. This figure could be in supplement but I feel maybe better at the beginning of the main figure to establish clearly how the data is generated.

2. The method utilizes day-old protoplasts for the experiment. While the original source tissue is leaf, it's not clear in the day-old protoplast if the cellular state and epigenome still retains a "leaf-like" properties. A comparison of RNA-seq and ATAC-seq between the leaf source tissue and leaf protoplast would be helpful to provide insight into how much/little has changed.

3. Not enough is presented on the QC of the ChIP data. Fig 1, Sup Fig 1 and text should have more information regarding:
a. Reproducibility between ChIP for the same factor. Sup Fig 1a kind of has this information but it's not possible to read the way it is set up. A table and possibly a plot showing correlation of all duplicated ChIP would be valuable.
b. It would be good to make it clear quality of the full set of ChIP-seq as presumably some are better than others. What is the Fraction Reads in Peak (FRIP score) and other relevant ENCODE metrics (fold enrichment etc)? Also, what is the cutoff to exclude a ChIP-seq from further analysis?
c. Provide a screen shots of TFs in a genome browser. This would be very helpful to have a dozen or so TFs from different families in a browser to get a sense of the quality of the ChIP-seq (ie are the peaks strong and sharp, or broad and only slightly above background, do they all overlap or is there clear shift between factors suggesting they are binding to distinct locations). Fig 5b does show a region of the genome with peaks but it is only three families and is zoomed out so it is hard to tell if the peaks are shifted relative to each other.

4. Sup Fig 1a can be improved and needs some additional explanation in the text.
a. What is that correlation block in the bottom of Sup Fig 1a. It appears to be mix of different families which is somewhat surprising.
b. The TF family legend is not readable. Use a larger pallet of colors not just blue-to-yellow.
c. It would be nice to see and additional figure like Sup Fig 1a that is order by family. Paralogs with 70% sequence similarity will target very similar motifs in vitro (doi:10.1016/j.cell.2014.08.009). Is this true in vivo? It doesn't appear this is the case in Sup Fig 1a as each factor seems to only correlate with itself closely and family TF members don't appear to cluster.

5. The heavy emphasis on network may miss opportunity to explore individual TF and TF families in more detail. In general, the text and analysis treat the set of TFs collectively even though there is likely to be huge degree of variation in properties of this large and diverse superfamily of genes. A lot of basic questions regarding TF family and individual TF trends could be explored and would be very interesting

a. What is the distribution relative to distal-promoter-5'UTR-gene body-3'UTR look like? Likely some TF/TF families are enriched in distinct regions. Can this data identify TFs that are primarily promoter active while others that may be more distal (possibly enhancer regions)?
b. Are there TFs that are not enriched in ATAC-seq regions, possible pioneer factors.

c. Do specific TF/TF families associate with specific epigenetic marks that can be recovered from literature (DNA methylation, H3K4me3, H3K27). If space is an issue, some of Fig 2 could be moved to supplement. It's nice to see that GWAS, eQTL etc enriched at TF sites, but is more confirmatory of what one would expect TFs and may not have much more to expose beyond that.

6. In Fig 5d there is a clade of TFs in the dendrogram that show very high co-binding with almost all TFs. What are the TFs in this clade? Is the paragraph starting at line 304 describing this set? I'm left wondering if having at set of TFs that appear to co-bind with nearly everything may be an artifact of the analysis.

Minor
- Sup Fig1 c, scale is much larger than displayed data upper range.
- Sup table 3 . the contents of the "short name" field doesn't seems to match to the first two fields. In first row it is C2H2-Col and Col8 but then wrky94 for shortname.
- Line 58, "Despite TF binding information is crucial…". May be better to say "Despite TF binding information being crucial…"
- Line 177 "We reshaped the ChIP-seq into a graph". Not sure that "reshape" is the correct word to use here.
- Line 229 "and are large to be" should be "and are too large to be"
- Line 300, typo, "and the its top three", "the" should be removed.

Reviewer #3 (Remarks to the Author):

The textbook view of relatively simple cis-regulatory control of eukaryotic gene expression has been widely discarded. The "real world" is tremendously more complex, with multiple transcription factors, enhancers, and other factors like nuclear localization interacting in a complex way to get transcription going for particular genes - more often than not from multiple transcription initiation sites. It seems almost hopeless to comprehend such complexity. And yet, each developing seed, each functional leaf, each instance of flowering (to stay with the plant examples) gives evidence that life solves such complex regulatory tasks, precisely.

The authors take an undaunted approach towards understanding the underlying gene regulatory networks. While not novel in approach, they describe the first large scale analysis of genome-wide transcription factor binding and network representation for maize, with comparative evaluation to other grains as well as Arabidopsis.

This is a stimulating paper with lots of interesting leads to follow for the astute reader. Without detailed follow-up, it is difficult to assess the robustness of the authors' statistical analyses. For example, is it really hard to fit a power law function to a biological network? All of biology is rife with diversity. So, yes, some transcription factors will bind to lots of places, others to few.

While interesting as is, the paper would greatly benefit from meticulous editing, not just for proper English, but also for content of statements. To illustrate, examples in order of appearance:

line 18 "underlying essential and complex functionalities" - what exactly would that mean? Are non-essential and simple functionalities really regulated differently?

line 20 "too few in number" - not accurate; in principle, one publication could give all the answers

line 22 "The resulted network" - not proper English

line 26 "were used to TF sequence preferences" - not a sentence

lines 52/53 "are located in the non-coding regions that has yet been functionally annotated" - presumably this is meant to read " that have yet to be functionally annotated?"

The above listing is by no means exhaustive. Every sentence should be checked.

Overall, a nice report of a lot of work that should push our understanding of transcription initiation in plants.

Volker Brendel

Reviewer #1 (Remarks to the Author):

Overall, this is a very interesting set of experiments that have been clearly described and fairly interpreted. The collection of ChIP-seq experiments provide a substantial new body of empirical data that will be very useful to the plant research community. The additional analyses support the validity of the data, and provide additional support for combinatorial models of regulation of transcription.

We thank the reviewer for the comment. It is our goal to make our dataset and derived models accessible to the community.

I have only two scientific comments. First, aside from the eQTL analysis and perhaps some co-expression analysis, it's not clear how extensively expression data has been used to interpret these ChIP-seq results on a genome wide scale, which would have the potential to provide some additional biological insight.

In our original version, we didn't include analysis using co-expression data. For the maize community several expression atlas have already being extensively used for the building of co-expression network (early ones with microarray, and later with RNA-seq data). Second, we wanted to complement the current maize gene regulatory prediction (based on expression data) using an orthogonal approach, that could eventually be used to associate *trans*-regulators with *cis*-variation, hence we mainly focused on genetic data to interpret the ChIP-seq results in the paper.

Relatedly and more specifically, the use of GRNs with conditional expression analysis may be informative. It would also potentially help to improve GRNs with other TFs not included in this dataset to compare their empirical results with GRNs that were constructed naive of the ChIP datasets for these 104 TFs.

We agree with the reviewer. GRNs based on co-expression are relatively easy to construct as it requires RNA-seq data which is easy to generate at scale than ChIP-seq data. However, co-expression doesn't provide enough information to distinguish whether these genes share the same or similar regulators, or if the TF is directly or indirectly controlling these genes.

We have compared our ChIP-seq based TF to target gene prediction with those derived from a co-expression GRN using 3 published maize RNA-seq datasets (different tissue: Walley 2016; leaf sections: Wang 2013; seed germination stage: Liu 2013). From the result, we found that the expression correlation of TF to target gene is significantly higher than TF to non-target. We have now added the following sentence to the result in page 9 line 228: "We overlaid our TF to target gene pairs with those inferred by previous co-expression analysis, and found that ChIP-seq identified gene pairs have higher co-expression correlation than the control (P-value < 2.2e-16)."

Also, I note the following grammatical/typographical issues:
P1 line 26 missing word:
complex agronomic traits. Machine-learning analyses were used to TF sequence preferences, and
line 42, eukaryotes?
unicellular model for eukaryote,
line 57 unnecessary comma
elements in the accessible chromatin ,and the TFs that bind to them
line 58 grammar…despite the fact that? Use "Although TF binding information…."?
Despite TF binding information is crucial for understanding how genes are regulated,
Line 60 unnecessary article "…express epitope fusion proteins" rather than "…the epitope fusion protein"
Line 63 should be "that are expressed" because talking about 104 TFs (plural)
Line 69 Use of instead of use
Line 71 perform ChIP-seq not performed
Typo on Manhattan Plot (Fig 2d) GAWS instead of GWAS
Line 175 I believe network should be plural in each usage (networks).

Thanks for the comments, we re-read the paper and have now corrected the errors.

Reviewer #2

The authors developed a high-throughput ChIP-seq protocol for Maize leaf protoplast and applied it to produce 218 ChIP-seq experiments for 104 TFs expressed in Maize leaf. They show that TF peaks are enriched in open chromatin, GWAS, and eQTL regions, and that there is a reduction in sequence variants in Maize strains at TF peak centers. Their primary use of the data set is for network analysis. First, they describe global properties of network modules, and then go into greater detail exploring TF modules implicated by their analysis to be involved in photosynthesis including mutant analysis of on factor. Finally, using ML they predict TF co-binding partners.
Overall it is a good study consisting of a new high-throughput approach for plant ChIP-seq, a major laboratory feat to perform 218 TFs, and a valuable resource for the community. The analysis is good but could be improved.
The primary issues I had is that they do not provide sufficient QC analysis of the ChIP, some figures can be improved for greater clarity and additional explanation of results is warranted in some cases, and more space could be provided for analysis of TF family/individual TF properties. Of these, better QC analysis of the ChIP-seq is most critical as it is difficult as it is presented to judge the data quality making it harder to fully assess the quality of the analysis and the resource.

1. The authors provide a very detailed protocol in the supplementary material, but the manuscript would benefit from a little more detail in the main text and figure to accompany it. This figure could be in supplement but I feel maybe better at the beginning of the main figure to establish clearly how the data is generated.

We thank to the reviewer for the suggestion. We have now added two paragraphs at the beginning of the result about how the protoplast transformation and ChIP-seq were performed (page 3) and how the data analysis and QC were done (page 4).

2. The method utilizes day-old protoplasts for the experiment. While the original source tissue is leaf, it's not clear in the day-old protoplast if the cellular state and epigenome still retains a "leaf-like" properties. A comparison of RNA-seq and ATAC-seq between the leaf source tissue and leaf protoplast would be helpful to provide insight into how much/little has changed.

Thank you for the suggestion, the chromatin accessibility profiles are indeed very similar between maize leaf and the protoplast we used. We have previously compared the epigenome (DNA methylation and open chromatin) of the maize leaf protoplast with other tissues, and found that they are very similar (Dong et al., 2017 and 2020 PMID: 29175436 & 30920762). In terms of gene expression, RNA-seq data from our leaf mesophyll protoplast and the published mature leaf data (PMID: 27540173) has a Pearson correlation of 0.6289, significantly higher than those with other tissues (e.g. protoplast vs root is 0.0352).

3. Not enough is presented on the QC of the ChIP data. Fig 1, Sup Fig 1 and text should have more information regarding:
a. Reproducibility between ChIP for the same factor. Sup Fig 1a kind of has this information but it's not possible to read the way it is set up. A table and possibly a plot showing correlation of all duplicated ChIP would be valuable.

We have now added a paragraph to the result section in the main text to summarize the QC result (page 3). We followed the ENCODE2 pipeline, which used SPP for individual ChIP-seq peak calling and the indices of reproducibility (IDR $\leq$ 0.01) to generate the final high confident peaks from the two biological replicates. Following on the reviewer suggestion, we have added a Supplementary Table 4 showing Pearson correlation between all ChIP-seq libraries. Correlation plot in Sup Fig. 1 has been revised to show the correlation of 28 ChIP-seq of 14 TFs in the EREB family, which is the largest TF family in our 104 TF collection.

b. It would be good to make it clear quality of the full set of ChIP-seq as presumably some are better than others. What is the Fraction Reads in Peak (FRIP score) and other relevant ENCODE metrics (fold enrichment etc)?

We added a Supplementary Table 2 showing the quality matrices generated by the ENCODE2 SPP-IDR pipeline for each ChIP-seq library. Overall, we followed the recommendations for using SPP-IDR for ChIP-seq data with replicates. The SPP-IDR approach uses the NSC/RSC for QC instead of FRIP which is used as a QC for calling peaks with MACS2, and doesn't use the IDR to identify reproducible peaks between replicates. The ENCODE2 paper showed that the FRIP and NSC/RSC values are highly correlated and NSC/RSC is preferred as they are less sensitive to peak calling bias (Landt et al., 2012. PMC: 3431496). All the peak calling results for the 104 TFs, including signal/fold enrichment etc, have been uploaded to GEO.

Also, what is the cut-off to exclude a ChIP-seq from further analysis?

We rejected ChIP-seq with NSC ≤ 1.05 and RSC ≤ 0.8 as recommended by the ENCODE2, and retained 217 ChIP-seq libraries, the QC result is in Supplementary Table 2. The raw read and peak calling results have been deposited in NCBI SRA and GEO.

c. Provide a screen shots of TFs in a genome browser. This would be very helpful to have a dozen or so TFs from different families in a browser to get a sense of the quality of the ChIP-seq (i.e., are the peaks strong and sharp, or broad and only slightly above background, do they all overlap or is there clear shift between factors suggesting they are binding to distinct locations). Fig 5b does show a region of the genome with peaks but it is only three families and is zoomed out so it is hard to tell if the peaks are shifted relative to each other.

We added a new Figure 2 in the main text showing a genome browser screen-shot with multiple TF ChIP-seq tracks in RAP2.7 gene locus and its well-known distal regulatory element *Vgt1* ~70kb upstream.

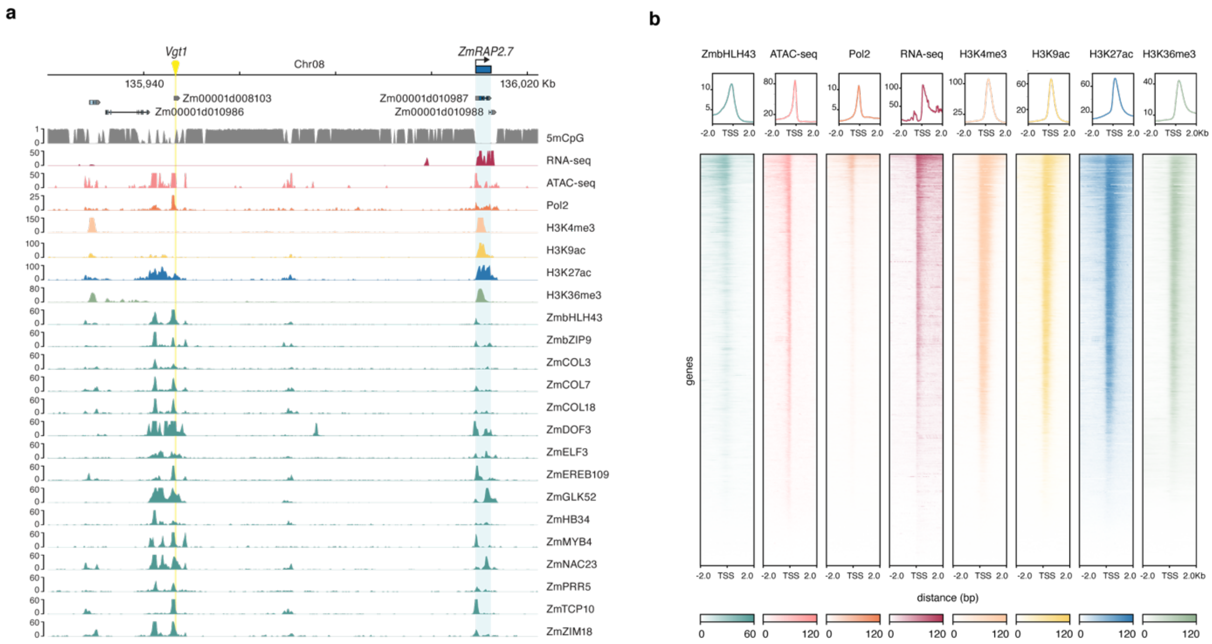

4. Sup Fig 1a can be improved and needs some additional explanation in the text.
a. What is that correlation block in the bottom of Sup Fig 1a. It appears to be mix of different families which is somewhat surprising.
b. The TF family legend is not readable. Use a larger pallet of colours not just blue-to-yellow.
c. It would be nice to see and additional figure like Sup Fig 1a that is order by family. Paralogs with 70% sequence similarity will target very similar motifs in vitro (doi:10.1016/j.cell.2014.08.009). Is this true in vivo? It doesn't appear this is the case in Sup Fig 1a as each factor seems to only correlate with itself closely and family TF members don't appear to cluster.

Thanks for the suggestion. Sorry for the crowded Sup Fig. 1a. We have now modified it to show the correlation of less TF (14 TF, 2 rep each in the EREB family, see below). All the correlation values from 104 TF have been added to Table S4. Yes, our k-mer correlation analysis shows that similar k-mer weight is obtained from TF members of the same family. In the same way, the PWM "motif" is similar among TF members from the same family. We added Sup Fig 1b to show PWMs "motif" of the EREB family.

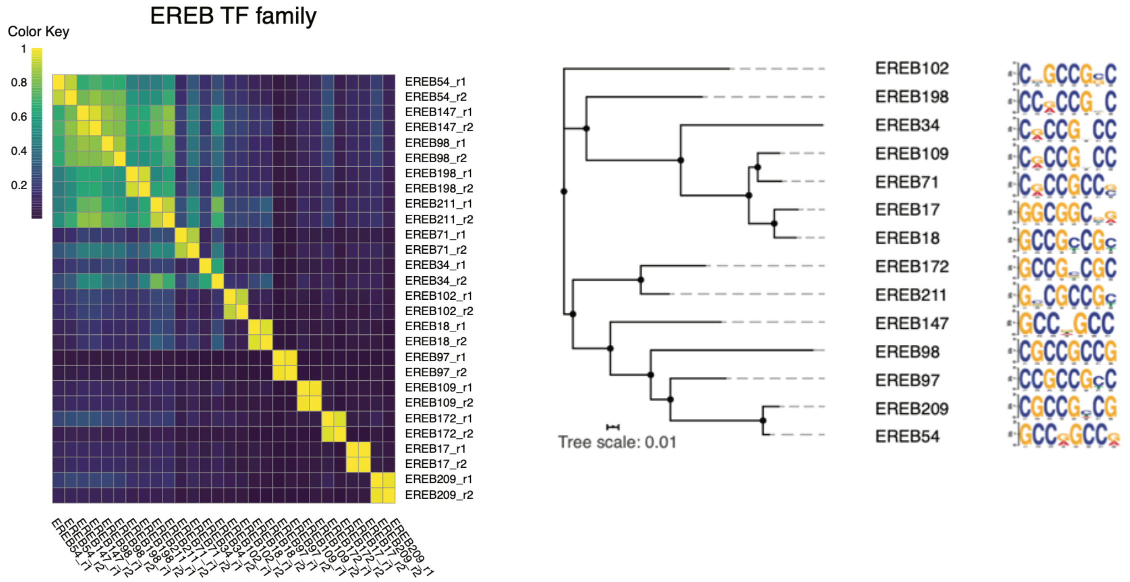

5. The heavy emphasis on network may miss opportunity to explore individual TF and TF families in more detail. In general, the text and analysis treat the set of TFs collectively even though there is likely to be huge degree of variation in properties of this large and diverse superfamily of genes. A lot of basic questions regarding TF family and individual TF trends could be explored and would be very interesting
a. What is the distribution relative to distal-promoter-5'UTR-gene body-3'UTR look like? Likely some TF/TF families are enriched in distinct regions. Can this data identify TFs that are primarily promoter active while others that may be more distal (possibly enhancer regions)?

We agree with the reviewer that each of the TFs represents its own interesting story. We hope the community will build on our global effort and explore more individual TF for their biological questions. We have calculated the ratio of distal to proximal binding for each TF. In a similar way we have run the co-localization model conditional to the peak location and calculated the differentiate score (DI) as in the Sup Fig. S6. We did not observed a significant enrichment in distal regions, all 104 TFs binding are most enriched in the gene proximal region. We speculate that the set of TFs we chosen (based on RNA-seq RPKM level in leaf) might be biased against those TFs that could binds to distal enhancer or regulate chromatin accessibility or associated with other epigenetic factors. Because the matured leaf tissue no longer require large-scale cell fate switches and differentiation.

b. Are there TFs that are not enriched in ATAC-seq regions, possible pioneer factors.

All 104 TF have binding enriched in the open chromatin (ATAC-seq) region. As above, the conditions to build our set of TFs might have prevented the sampling of pioneer factors. For instance, pioneer TFs would probably be expressed in the early stage during cell fate determination and probably in or near the meristem tissue. But all the 104 TFs we studied are those highly expressed in leaf, where there is no need for cell fate switch.

c. Do specific TF/TF families associate with specific epigenetic marks that can be recovered from literature (DNA methylation, H3K4me3, H3K27). If space is an issue, some of Fig 2 could be moved to supplement. It's nice to see that GWAS, eQTL etc enriched at TF sites, but is more confirmatory of what one would expect TFs and may not have much more to expose beyond that.

Our 104 TF list does not contain known 5mC-binder or histone modification binding TFs. As mentioned above, most of those TFs will not be highly expressed in a differentiated tissue like the mature leaf. We added a Fig. 2a showing varies epigenetic marks with TF binding in a well-known locus Vgt1. We have also added Fig 2b and Sup Fig 2 showing the DNA methylation and histone mod (H3K4me3, H3K9ac, H3K27ac and H3K36me3), as well as Pol2, RNA-seq track and ATAC-seq tracks. We now showed ZmbHLH43 (which is one of the TF that binds to *Vgt1* in Fig. 2a) as an example in Fig. 2b and Fig S2 (similar epigenome feature in its distal binding sites).

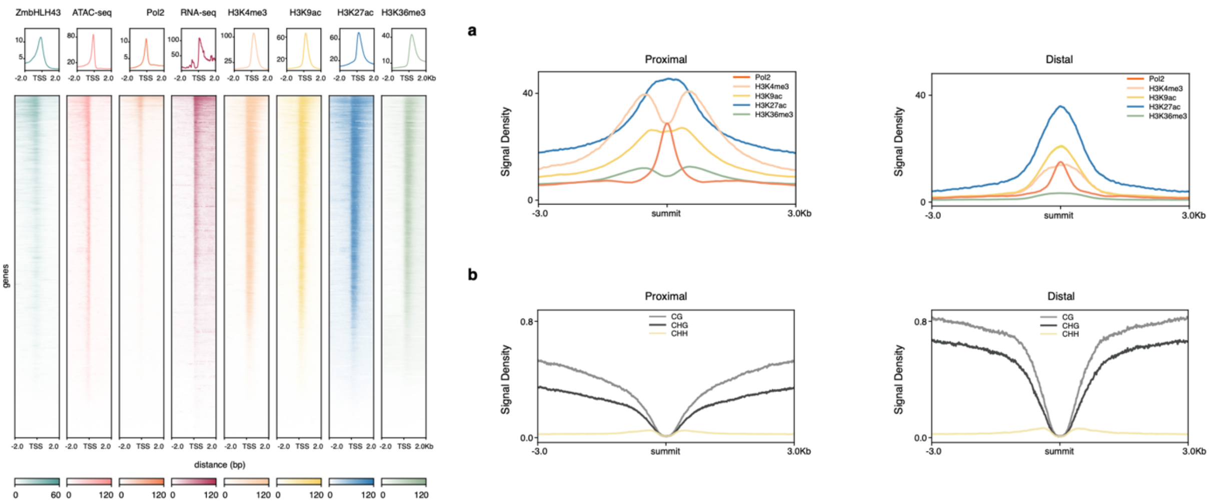

6. In Fig 5d there is a clade of TFs in the dendrogram that show very high co-binding with almost all TFs. What are the TFs in this clade? Is the paragraph starting at line 304 describing this set? I'm left wondering if having at set of TFs that appear to co-bind with nearly everything may be an artefact of the analysis.

We are sorry that this figure was not clearly explained and we have added a sentence to clarify it in page 14 Line 352. The figure doesn't show co-binding itself. It shows the results of the machine learning model to predict the binding of each TF from the co-localization with other TFs (in form of RI value of each TF as a partner in each TF's context). In brief, the RI value is the relative importance according to the model for a partner TF to explain the binding of a focus TFs. In an oversimplified case, if the partner TF-A co-localize with the focus TF-B in 30% of the peaks, but partner TF-C doesn't co-bind well with the focus TF-B, e.g. in more than 5% of the peaks, TF-A as partner will be considered highly explanatory of the focus TF-B, and will be assign a high RI value. But different TFs have different overlap in terms of binding, and hence the RI value could not be directly inferred as co-binding level. Our interpretation is that TFs with a high RI will be highly combinatorial. We found that TFs with high RI in several families (*i.e.,* bHLHs, ZIM, MYB, EREB, COL), so it is not a bias related to a single type of TF family. In addition, the TFs with high RI all passed the strict QC criteria as recommended by the ENCODE2 pipeline, and we didn't observed a departure in the number of peaks, or number of target genes from the remaining TFs, suggesting that it is not an artefact. Further on, the ENCODE used the same strategy to identify human TFs with high RI.

Minor
- Sup Fig1 c, scale is much larger than displayed data upper range.
- Sup table 3 . the contents of the "short name" field doesn't seems to match to the first two fields. In first row it is C2H2-Col and Col8 but then wrky94 for shortname.
- Line 58, "Despite TF binding information is crucial…". May be better to say "Despite TF binding information being crucial…"
- Line 177 "We reshaped the ChIP-seq into a graph". Not sure that "reshape" is the correct word to use here.
- Line 229 "and are large to be" should be "and are too large to be"

- Line 300, typo, “and the its top three”, “the” should be removed.

Thank you so much for spotting the err. We have now corrected those.

Reviewer #3 (Remarks to the Author):

The textbook view of relatively simple cis-regulatory control of eukaryotic gene expression has been widely discarded. The "real world" is tremendously more complex, with multiple transcription factors, enhancers, and other factors like nuclear localization interacting in a complex way to get transcription going for particular genes - more often than not from multiple transcription initiation sites. It seems almost hopeless to comprehend such complexity. And yet, each developing seed, each functional leaf, each instance of flowering (to stay with the plant examples) gives evidence that life solves such complex regulatory tasks, precisely.

The authors take an undaunted approach towards understanding the underlying gene regulatory networks. While not novel in approach, they describe the first large scale analysis of genome-wide transcription factor binding and network representation for maize, with comparative evaluation to other grains as well as Arabidopsis.

This is a stimulating paper with lots of interesting leads to follow for the astute reader. Without detailed follow-up, it is difficult to assess the robustness of the authors' statistical analyses.

For example, is it really hard to fit a power law function to a biological network? All of biology is rife with diversity. So, yes, some transcription factors will bind to lots of places, others to few.

It should not be hard to fit a power law function to a biological network. The same is true for other networks beyond biology such as the world wide web or social networks. However, a network must be “true” to obey this principle, drawing any network won’t recapitulate the universal architecture. For instance, the network presented in the manuscript, when compared with randomly rewired networks (even as we maintained the distribution of the in/out degree) shows an increase in modularity, which is also typical behaviour of true networks. In other words, if the data from which the network is drawn is randomly synthesized or flawed, we should not expect the resulting network to exhibit behaviours of a true network. In addition, if the sampling is not sufficient (e.g. with only 10-20 TFs ChIP-seq), one will not be able to see the topology and modularity.

While interesting as is, the paper would greatly benefit from meticulous editing, not just for proper English, but also for content of statements. To illustrate, examples in order of appearance:

line 18 "underlying essential and complex functionalities" - what exactly would that mean? Are non-essential and simple functionalities really regulated differently?

line 20 "too few in number" - not accurate; in principle, one publication could give all the answers

line 22 "The resulted network" - not proper English

line 26 "were used to TF sequence preferences" - not a sentence

lines 52/53 "are located in the non-coding regions that has yet been functionally annotated" - presumably this is meant to read " that have yet to be functionally annotated?"

The above listing is by no means exhaustive. Every sentence should be checked.

Overall, a nice report of a lot of work that should push our understanding of transcription initiation in plants.

Thank you for comments and suggestions. We have corrected those and polished the writing in the manuscript.

REVIEWERS' COMMENTS:

Reviewer #1 (Remarks to the Author):

This revision adequately addresses the concerns raised in my initial review.

Reviewer #2 (Remarks to the Author):

The figure and text changes have addressed my main concerns. This revised manuscript represents an important contribution to understanding plant gene regulatory network properties.

[Editor: Reviewer #3 states in Remark to Editor section that (s)he is satisfied with revision, but ask authors to edit English language before publication.]
